# Global Frequency Analyses of Canine Progressive Rod-Cone Degeneration–Progressive Retinal Atrophy and Collie Eye Anomaly Using Commercial Genetic Testing Data

**DOI:** 10.3390/genes14112093

**Published:** 2023-11-17

**Authors:** Jessica A. Clark, Heidi Anderson, Jonas Donner, Susan Pearce-Kelling, Kari J. Ekenstedt

**Affiliations:** 1 Department of Basic Medical Sciences, College of Veterinary Medicine, Purdue University, West Lafayette, IN 47907, USA; clark542@purdue.edu; 2 Wisdom Panel, Mars Petcare Science & Diagnostics, 00581 Helsinki, Finland; heidi.anderson@wisdompanel.com (H.A.); jonas.donner@wisdompanel.com (J.D.); 3 Wisdom Panel, Mars Petcare Science & Diagnostics, Portland, OR 97209, USA; susan@wisdompanel.com

**Keywords:** dog, vision, disease, optic, blind, geography, distribution, country, breed

## Abstract

Hundreds of genetic variants associated with canine traits and disorders have been identified, with commercial tests offered. However, the geographic distributions and changes in allele and genotype frequencies over prolonged, continuous periods of time are lacking. This study utilized a large set of genotypes from dogs tested for the progressive rod-cone degeneration–progressive retinal atrophy (prcd-PRA) G>A missense *PRCD* variant (*n* = 86,667) and the collie eye anomaly (CEA)-associated *NHEJ1* deletion (*n* = 33,834) provided by the commercial genetic testing company (Optigen/Wisdom Panel, Mars Petcare Science & Diagnostics). These data were analyzed using the chi-square goodness-of-fit test, time-trend graphical analysis, and regression modeling in order to evaluate how test results changed over time. The results span fifteen years, representing 82 countries and 67 breeds/breed mixes. Both diseases exhibited significant differences in genotype frequencies (*p* = 2.7 × 10^−152^ for prcd-PRA and 0.023 for CEA) with opposing graphical trends. Regression modeling showed time progression to significantly affect the odds of a dog being homozygous or heterozygous for either disease, as do variables including breed and breed popularity. This study shows that genetic testing informed breeding decisions to produce fewer affected dogs. However, the presence of dogs homozygous for the disease variant, especially for prcd-PRA, was still observed fourteen years after test availability, potentially due to crosses of unknown carriers. This suggests that genetic testing of dog populations should continue.

## 1. Introduction

With the advent of genetic testing for dogs, canine health care could begin to embrace preventative genetic screening; when tests became available for fully penetrant autosomal recessive diseases, breeders now had accurate tools to predict outcomes and carefully plan matings to avoid producing affected puppies. To date, over 320 Mendelian traits and conditions have had a likely causal variant(s) described [1,2] and some of these have been available as genetic tests for well over a decade. However, very few studies have examined results for such tests over time, reporting on the changing frequencies year by year, and their limited scope in terms of breed and geography provides only a narrow picture of true test adoption and response within breeds globally. 

In order to obtain a larger and more complete perspective, including the changing genotype and allele frequencies for numerous breeds over time and with a global context, data are best harnessed from commercial canine genetic testing providers. In the present study, we utilize current data from a large provider merged with historical testing data from a single genetic testing provider (OptiGen) that offered the examined tests nearly exclusively for the first ten years of their lifespan. The latter means that the captured picture (from 2004 or 2005 to 2013) essentially represents the global population of dogs being tested. Two ophthalmic diseases that fit this profile were selected for the present study: progressive rod-cone degeneration–progressive retinal atrophy (prcd-PRA) (OMIA 001298-9615) and collie eye anomaly (CEA) (OMIA 000218-9615).

PRA, affecting approximately one quarter of all dog breeds [3], is the canine equivalent of retinitis pigmentosa (RP) in humans [3]. RP, which affects one in 4000 people [4], consists of many different forms with more than 3100 mutations associated with non-syndromic RP alone [5]. Indeed, human RP-17 shares an identical causal mutation with canine prcd-PRA [6]. Prcd-PRA, one of 19 types of PRA listed in OMIA [1], is a progressive ocular disease characterized by the degeneration of the retinal rod and cone photoreceptors, leading first to night blindness and eventually complete blindness [6]. The prcd-PRA variant maps to canine chromosome 9 (CFA9) and consists of a missense mutation (c.5G>A) in *PRCD* [6], a retinal gene required for appropriate photoreceptor disc formation [7,8]. The missense variant results in the substitution of cysteine to tyrosine at *PRCD*’s second amino acid residue [6]; the loss of this particular cysteine results in the mislocalization of the PRCD protein thereby acting as a functional null mutation [8]. The mode of inheritance for prcd-PRA is single gene/Mendelian, and autosomal recessive, meaning that heterozygotes are healthy [3,9]. Because prcd-PRA is essentially fully penetrant, dogs homozygous for the variant allele will eventually go blind, although the age of onset can depend on breed background [3]. The prcd-PRA variant has been identified in 57 breeds and mixed breed dogs according to the published literature [10,11,12,13,14,15] plus an additional 22 breeds according to online sources [2,16,17,18,19,20] (Table S1); the wide breed representation suggests an ancient origin [15].

CEA is a hereditary ocular disease affecting multiple dog breeds, particularly herding breeds such as Collies and Shetland Sheepdogs. In contrast to prcd-PRA, CEA does not appear to have a synonymous human condition. CEA is non-progressive, very heterogeneous [21], and characterized mainly by choroidal hypoplasia (CH) and optic nerve head coloboma, which can occur separately or together [22]. Effects on a patient’s vision vary widely and can include blindness related to retinal detachment or intraocular hemorrhage [23], but the majority have minimal changes to vision [24]. CEA lesions are bilateral but rarely symmetrical [23]. The CH lesions consist of abnormalities in the retina or choroid, which lack pigment and can be identified in the first two to three months of a puppy’s life, although this can be difficult if the dog has a merle coat pattern [22]. Colobomas are more severe, consisting of pitting or excavations in the optic disc; depending on size, colobomas can lead to decreased vision [22,23]. The mode of inheritance for CEA is autosomal recessive with incomplete penetrance [25,26,27,28]. Fine-mapping studies utilizing multiple breeds identified a proposed CEA causal CFA37 variant consisting of a 7.8 kb *NHEJ1* intronic deletion that segregated with disease state [29]. Interestingly, although humans can develop macular colobomas [30], *NHEJ1* variants described in humans manifest as immunodeficiency, microcephaly, and growth delay [31,32], and the relationship between the 7.8 kb canine *NHEJ1* variant and non-ocular disease states (i.e., immunodeficiencies) is unknown [23]. The deletion variant has been identified in 24 breeds and mixed breed dogs in the scientific literature [12,13,29,33,34,35] to date, and in 14 more breeds according to additional online references [2,17,18,20,36,37] (Table S2). The majority of these breeds are in the herding group with a few exceptions; therefore, this allele likely predates the formation of herding breeds. Additional work is needed to determine if the outlier breeds signal an even more ancestral origin. Complicating the situation, subsequent work now indicates that the *NHEJ1* deletion may actually be linked to the true causal mutation, as the deletion does not segregate with coloboma or CH in some cases [24,38]. There is also debate whether CH and coloboma result from the same genetic cause [39] or whether additional, as yet unidentified, genetic variants are involved. A recent abstract suggests CEA colobomas are inherited in a complex manner and that multiple loci are likely involved [40]. In the absence of other known risk variants [38], the *NHEJ1* deletion currently remains the only variant tested in genetic panels for CEA risk.

Laboratories providing prcd-PRA and CEA testing have supplied a snapshot of the prevalence for these conditions, both geographically and by breed. A large-scale study (*n* = 101,427 canids) tested for the CEA deletion and reported a disease-associated allele frequency of 1.600% and 1.080% among mixed-breed and purebred dogs, respectively; this ranks CEA as the 5th and 6th most prevalent allele in those populations [13]. The same study determined prcd-PRA allele frequencies in mixed-breed dogs and purebred dogs of 3.418% and 1.746%, respectively, which placed prcd-PRA as the 3rd and 2nd most prevalent allele among those respective populations [13]. Other studies using various breeds in different countries have calculated the allele frequency of the CEA deletion at 0.43–79.7% and the prcd-PRA variant at 1.2–45% [14,33,34,41,42,43,44,45,46,47]. One study tracked disease-causing variants over time in eight breeds for eight single-gene disorders, one of which was prcd-PRA [46]. This investigation was limited by breed to Labrador Retrievers and Cocker Spaniels and geographically to the United Kingdom (test results reported to the Kennel Club). They observed the prcd-PRA missense variant frequency decrease substantially (by about 90%) in the 8–10 years after the mutation’s publication [46]. 

While previous work has been instrumental in beginning to define disease prevalence and the changes in allelic and genotypic frequencies over time, these studies tend to be limited in time, breed, or geography [10,13,14,41,42,43,45,46,47]. In order to report a deeper and wider understanding of genetic test adoption and response within multiple breeds at a global scale, the objective of this study was to utilize commercial genetic testing data from a global canine population spanning 15 years and many breeds for both prcd-PRA and CEA. We hypothesized that over the time of test availability, there would be a significant decline in the frequency of homozygous-affected genotypes, heterozygous carrier genotypes, and the alternate, disease-associated allele due to selection pressure by breeders based on DNA testing results. We expected to see this decline at the global level, within individual countries, and within breeds.

## 2. Materials and Methods

### 2.1. Samples

Data for this study comprised genotype status for prcd-PRA (*n* = 86,667 dogs) and CEA (*n* = 33,834 dogs) tested between the years 2004–2019 at OptiGen, LLC (acquired by Mars Petcare in 2018), a commercial genetic testing company. Each dog was also assigned an owner-reported breed or breed mix and country. No samples were duplicated within each disease; however, information was not available for evaluating the overlap of the same dog tested for both diseases. The prcd-PRA dataset represented 61 breeds and breed mixes and 74 countries, and the CEA dataset represented 21 dog breeds and breed mixes and 59 countries (Tables S3 and S4). Each year represents a full calendar year’s worth of data, i.e., the first year (2004 for pcrd-PRA and 2005 for CEA) begins with data from 1 January, and the 2019 data for both ends with 31 December. From 2004 to 2013, OptiGen was the sole provider of prcd-PRA and CEA testing, and for these years this dataset comprises a relatively complete global testing picture. Of note, another company began offering these tests in Asian and Pacific countries, Australia and New Zealand in 2006, so 2006–2019 are unlikely to be fully representative for these countries.

All samples were voluntarily submitted by pet owners to OptiGen between 2004–April 2019 for DNA testing as a paid commercial service. At the time of submission, the owner provided consent for the use of samples in scientific research. Samples from blood and cheek cells (via buccal swab) underwent DNA extraction according to standard protocols, and DNA from semen samples was extracted using the Qiagen QIAmp DNA mini kit cat# 51306 (Valencia, CA, USA) protocol. The samples were individually tested through the beginning of 2019 [6,14,29] and thereafter were included on a proprietary custom panel to identify the number of prcd-PRA or CEA variant alleles present in each dog.

### 2.2. Statistical Analyses

For each breed and each country in any given year, genotype frequencies were calculated by dividing test status (i.e., genotype; normal/carrier/affected, translating to homozygous reference, heterozygous, and homozygous variant) by total number of dogs tested within the given year. Allele frequencies for reference and alternate (disease-associated alleles were calculated for each year, under the assumption of one alternate (disease-associated) allele per carrier and two per affected dog. Frequencies were then plotted against the year to illustrate changes over time for chosen dog breeds. Breeds emphasized in this paper have a minimum of 50 dogs tested in at least half of the years (eight years) observed for each disease (prcd-PRA: 18 breeds, CEA: 5 breeds) (Table S5).

To determine if there was a significant difference in genotype and allele frequencies from the beginning to the end of the recorded timeframe for prcd-PRA (2004–2019) and CEA (2005–2019), chi-square goodness-of-fit tests were conducted for both diseases under the null hypothesis that there was no change in genotype frequencies between the first and last year of data. The significance for all statistical analyses was set at α ≤ 0.05. To investigate the effect of different variables on the probability of a dog having a particular disease genotype while incorporating all years of data, (1) logistic regression models (LRM) were used to observe the log odds of a dog having “disease-positive” (homozygous-affected) status as opposed to “disease negative” status (heterozygous or homozygous wild type) and (2) a multinomial regression model (MRM) was used to observe the log odds of a dog being identified as having homozygous-affected or heterozygous carrier genotype, compared with homozygous wild type as the reference or baseline. For every component investigated, both an LRM and MRM with the same variables were included and every LRM and MRM model was weighted by a count of samples to address sample size differences between variables (e.g., between different breeds, between different countries, and between different years).

The log odds of disease status for any given dog, using a logistic regression model, was modeled as:

$$\log\left(\frac{p_{positive\;for\;disease}}{p_{negative\;for\;disease}}\right)=\beta_{0}+\beta_{1}\left(variable\;1\right)+\ldots +\beta_{n}\left(variable\;n\right)$$

The log odds of genotype status for any given dog, relative to homozygous wild type, using a multinomial regression model, was modeled as:

$$\log\left(\frac{p_{homozygous\;variant}}{p_{homozygous\;wild-type}}\right)=\beta_{0}+\beta_{1,hom}\left(variable\;1\right)+\ldots +\beta_{n,\;hom}\left(variable\;n\right)$$

$$\log\left(\frac{p_{heterozygous}}{p_{homozygous\;wild-type}}\right)=\beta_{0}+\beta_{1,het}\left(variable\;1\right)+\ldots +\beta_{n,het}\left(variable\;n\right)$$

where for both models, $\beta_{0}$ represents the intercept term, $\beta_{n}$ is the coefficient for the variables included in the model, and the variable itself is the categorial or numerical value provided to a dog as it relates to the investigated component.

To determine the effects of year and breed on log odds, the models included the following variables: year of testing (continuous), breed (categorical), and country (categorical). To determine the effects of popularity on log odds, the models included year and breed ranking (continuous) according to the American Kennel Club (AKC) registration numbers in 2004 (prcd-PRA) and 2005 (CEA) where, as the dog breed became more popular, they experienced a greater number of new registrations, and their overall rank became closer to one. For all models, in order to create adequate sample sizes and decrease confusion, samples identified as having an owner-labeled breed designation of “FarmCollie”, “Collie”, “Smooth Collie”, “Rough Collie”, and “FamCollie” were all considered under the umbrella of “Collie”. The same breed rank analyses were also carried out for the year 2019 AKC rankings (last year included in the dataset) in order to determine if trends remained the same over time.

## 3. Results

### 3.1. Data Distributions and Disease Comparisons

**Figure 1:**
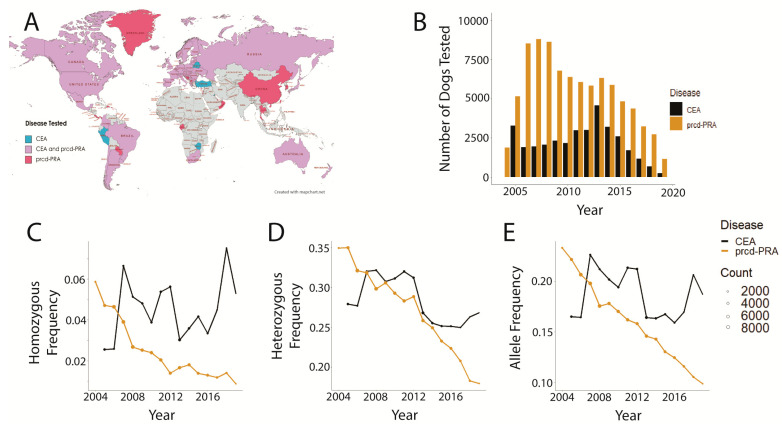
Distribution of genetic tests and trend comparisons between prcd-PRA (2004–2019) and CEA (2005–2019). (**A**) Owner-reported geographic origin of samples submitted for prcd-PRA and CEA genetic testing. The gray color indicates no samples were received from that country. This image does not include Bermuda, French Polynesia, Gibraltar, Macau (prcd-PRA), Liechtenstein and Netherlands Antilles (CEA and prcd-PRA), and Bouvet Island and Norfolk Island (CEA). (**B**) Time distribution of genetic tests. (**C**) Frequency of homozygous variant genotype over time. Point size indicates the number of dogs tested that year. (**D**) Frequency of heterozygous genotype over time. Point size indicates the number of dogs tested that year. (**E**) Frequency of disease-associated allele over time. Point size indicates the number of dogs tested that year.

Genetic testing spanned all seven continents (Figure 1A), although Antarctica was represented by only one sample from Bouvet Island (CEA) and the majority of samples originated from North America and Europe (98.29% of prcd-PRA samples and 96.14% of CEA samples were from North American and European countries). The samples originated from a total of 82 countries, 50 of which contributed to both disease datasets, while 24 were unique to the prcd-PRA dataset, and 8 were unique to the CEA dataset (Table S3). The annual prcd-PRA test distribution demonstrated bimodal distribution with a peak of tests in 2007 and a smaller peak in 2013 (Figure 1B). The annual CEA test distribution was more unimodal, outside of a spike in 2005, with the largest influx of tests conducted in 2013 (Figure 1B). For both tests, the number of tested dogs tapered after 2013. Trends in disease genotype and allele frequency for prcd-PRA aligned with hypothesized downward trends. However, CEA did not conform to the hypothesized downward trends, instead, remaining either unchanged or even potentially increasing (Figure 1C–E) when inspected visually.

A chi-square goodness-of-fit test comparing the genotype frequencies in the first year of data (2004 for prcd-PRA, 2005 for CEA) with the final year of data (2019 for both diseases) showed a statistically significant difference (α ≤ 0.05) in genotype frequencies for both diseases (prcd-PRA *p* = 2.7 × 10^−152^; CEA *p* = 0.023244) (Table 1).

Logistic regression and multinomial regression models including the effects of year, breed, and country produced negative estimates of the effect of year (year coefficient, or the coefficient assigned to the variable of year in regression models) on log odds for either a homozygous-affected or heterozygous carrier genotype (Table 2). This means that all other variables were the same (same breed of dog and same country); as time moves forward, the overall log odds of a dog being affected or a carrier for either disease decreases according to the models. In all cases, the magnitude of the effect of year on log odds is five times greater for prcd-PRA than for CEA, suggesting that for every year progression through time, the log odds of a dog having an affected or carrier genotype decreased five times faster for prcd-PRA than for CEA.

**Figure 2:**
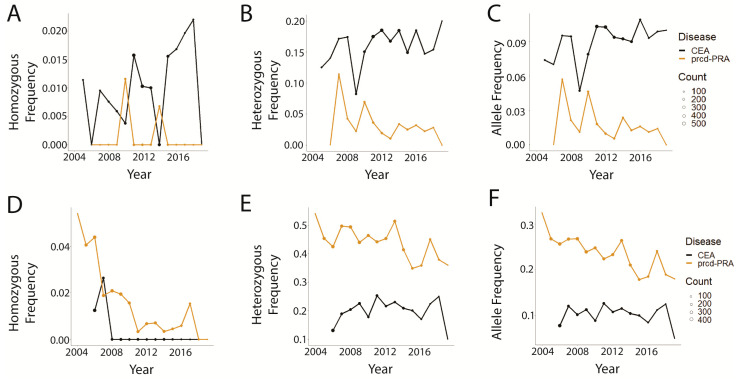
Genotype and allele frequencies over time of two dog breeds tested for both prcd-PRA and CEA. Point size indicates the number of dogs tested that year. (**A**) Australian Shepherd homozygous variant genotype, (**B**) Australian Shepherd heterozygous genotype, (**C**) Australian Shepherd disease-associated variant allele, (**D**) Nova Scotia Duck Tolling Retriever homozygous variant genotype, (**E**) Nova Scotia Duck Tolling Retriever heterozygous genotype, (**F**) Nova Scotia Duck Tolling Retriever disease-associated variant allele.

Next, among breeds tested for both disease variants, two representative breeds, Australian Shepherds (AS) and Nova Scotia Duck Tolling Retrievers (NSDTR) were selected to demonstrate frequency changes over time. Disease trends in AS (Figure 2A–C) and NSDTR (Figure 2D–F) showed similar patterns between the two breeds, with the prcd-PRA genotype and variant allele frequencies declining at a steeper rate compared with those of CEA. The latter exhibited a consistent or slightly increasing trend, with the exception of efficient eradication of the homozygous variant genotype in NSDTRs (Figure 2D). CEA-affected and carrier genotypes were generally more prevalent than prcd-PRA-affected and carrier genotypes in AS (Figure 2A–C), while the opposite was true in NSDTR (Figure 2D–F).

**Figure 3:**
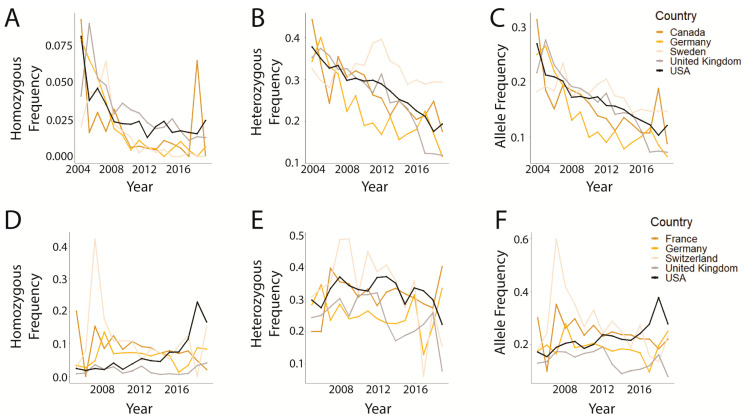
Genotype and allele frequencies in the top five tested countries for prcd-PRA (2004–2019) and CEA (2005–2019). (**A**) prcd-PRA homozygous variant genotype, (**B**) prcd-PRA heterozygous genotype, (**C**) prcd-PRA disease-associated variant allele, (**D**) CEA homozygous variant genotype, (**E**) CEA heterozygous genotype, (**F**) CEA disease-associated variant allele.

Amongst all represented countries, the number of dogs tested in any individual country ranged from 1 to 35,111 (United States of America) and 1 to 7221 (United States of America) for prcd-PRA and CEA, respectively (Table S3). The top five countries that submitted samples for prcd-PRA testing were Canada, Germany, Sweden, the UK, and the USA representing 75.73% of all samples; all trends showed a declining pattern (Figure 3A–C), except for Sweden, which demonstrates a relatively consistent frequency, peaking at 2012 (Figure 3B). The top five countries that submitted samples for CEA testing were France, Germany, Switzerland, the United Kingdom (UK), and the United States of America (USA) representing 64.88% of all samples; all five showed decreasing or consistent frequency trends (Figure 3D–F) except for the USA, which exhibited a striking increase in frequency of homozygous variant dogs (Figure 3D) and the disease-associated allele (Figure 3F). 

**Figure 4:**
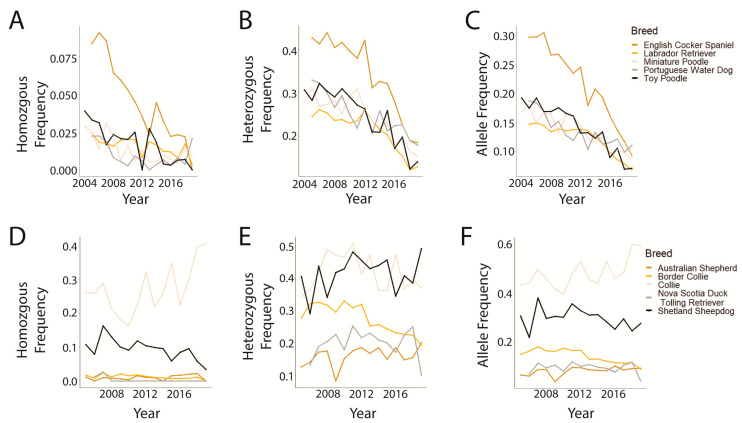
Genotype and allele frequencies in the top five tested breeds for prcd-PRA (2004–2019) and CEA (2005–2019). (**A**) prcd-PRA homozygous variant genotype, (**B**) prcd-PRA heterozygous genotype, (**C**) prcd-PRA disease-associated variant allele, (**D**) CEA homozygous variant genotype, (**E**) CEA heterozygous genotype, (**F**) CEA disease-associated variant allele.

Our dataset represented 67 distinct owner-reported dog breeds and breed mixes with 61 represented in the prcd-PRA dataset and 19 in the CEA dataset; 13 breeds are represented in both datasets (Table S4). Of these 67 breeds and breed mixes, 52 had the disease-associated allele for at least one of the two diseases tested (Table S6). The number of dogs tested in an individual breed ranged from 1 to 25,382 (Labrador Retriever) for prcd-PRA and 1 to 20,588 (Border Collie) for CEA (Table S4). The genotype and allele frequencies for the top five breeds tested for prcd-PRA (English Cocker Spaniel, Labrador Retriever, Miniature Poodle, Portuguese Water Dog, and Toy Poodle) all showed distinctly decreasing trends (Figure 4A–C). The top five breeds tested for CEA were Australian Shepherd, Border Collie, Collie, Nova Scotia Duck Tolling Retriever, and Shetland Sheepdog; genotype and allele frequencies remained relatively consistent or decreased slightly, except for the homozygous variant genotype and disease-associated allele frequency in the Collie and the heterozygous genotype in the Shetland Sheepdog, all three of which increased over time (Figure 4D–F). 

### 3.2. Trends in prcd-PRA

Forty-one breeds and breed mixes in our prcd-PRA dataset were genotyped for having at least one copy of the *PRCD* variant (Table S6). A statistically significant effect (α ≤ 0.05) on the probability of prcd-PRA genotype was shown for twenty-two of the breeds/mixes tested using logistic regression modeling (Table 3 and Table S7). Of these, eighteen breeds met the minimum of 50 dogs tested in at least half of the years, and are further examined in this paper. The largest frequency of the prcd-PRA allele (0.425 in 2004) was observed in American Eskimo Dogs, while it was Swedish Lapphunds that exhibited the greatest log-odds of a homozygous-affected or heterozygous genotype for prcd-PRA according to all models (Table 3). Overall, Australian Shepherds had the least log odds (i.e., of all breeds focused on in this study, Australian Shepherds are the breed least likely to have or carry for prcd-PRA at a statistically significant level) (Table 3).

**Figure 5:**
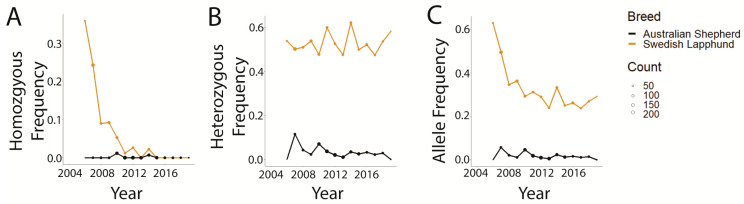
Genotype and allele frequencies in Swedish Lapphunds and Australian Shepherds tested for prcd-PRA (2004–2019). Point size indicates the number of dogs tested that year. (**A**) Homozygous variant genotype. (**B**) Heterozygous genotype. (**C**) Disease-associated variant allele.

Graphical comparisons of the breeds with the greatest and lowest likelihood of homozygous-affected and heterozygous genotypes (Table 3), Swedish Lapphund and Australian Shepherd, respectively, demonstrated that the Swedish Lapphund exhibited much higher genotype (affected and carrier) and disease allele frequencies than Australian Shepherds overall (Figure 5A–C). The only time this was not the case was in the frequency of homozygous-affected genotypes from 2015 forward, where both Swedish Lapphund and Australian Shepherd frequencies were zero (Figure 5A).

**Figure 6:**
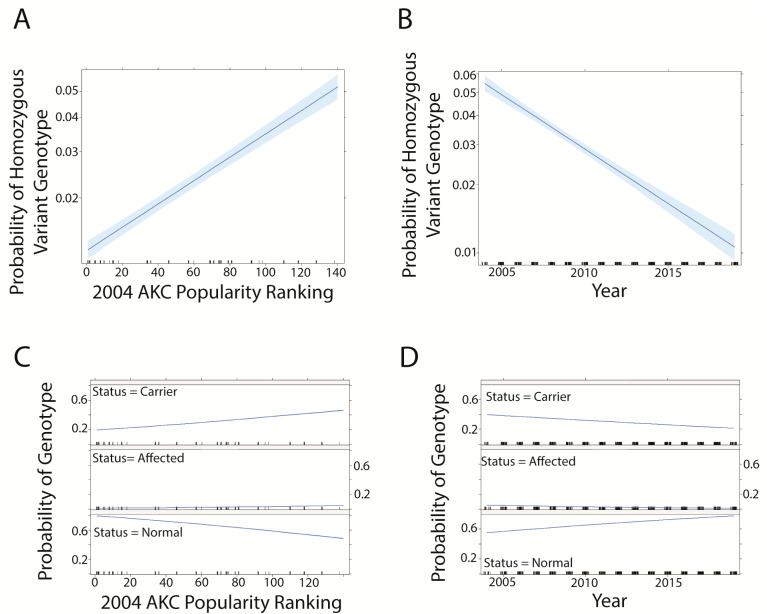
Effect of breed popularity according to AKC ranking by number of new registrations in 2004 based on probability of prcd-PRA genotype in models including breed rank and year. Blue shading indicates a 95% confidence interval. The most popular breed = rank #1. (**A**) Effect of breed rank (logistic regression model). (**B**) effect of year (logistic regression model). (**C**) effect of breed rank (multinomial regression model). (**D**) effect of year (multinomial regression model).

Interestingly, dog breeds with a lower popularity ranking according to 2004 AKC registration counts (Table S8) showed a greater probability of having a homozygous alternate (variant) or heterozygous genotype than breeds of greater popularity (Figure 6A,C). As time progresses, the probability of a dog having either genotype decreases (Figure 6B,D). 

**Figure 7:**
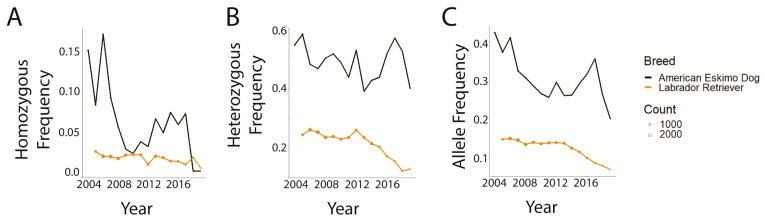
Global prcd-PRA genotype and allele frequencies of Labrador Retriever and American Eskimo Dog according to the 2004 new registration AKC ranking. Point size indicates the number of dogs tested that year. (**A**) Homozygous variant genotype. (**B**) heterozygous genotype. (**C**) disease-associated variant allele.

In 2004, according to AKC registration counts, the Labrador Retriever was the highest ranked (rank = #1), while the Field Spaniel was the lowest (Rank = #138). A graphical comparison of Labrador Retrievers (ranked number 1, i.e., most popular) and American Eskimo Dogs (ranked 110), selected as breeds with very different 2004 popularities, demonstrated that both breeds had a generally decreasing trend in the frequencies of the affected genotype, carrier genotype, and the overall disease-associated allele frequency (Figure 7). Frequencies for the American Eskimo Dog remained higher in magnitude at all time points, with the exception of the homozygous variant genotype frequency of 2018–2019 where they dropped below those of the Labrador Retriever.

To investigate other characteristics that may have an impact on genotype and phenotype likelihoods, regression analyses were performed considering the AKC group (model variables: AKC group + year), Fédération Cynologique Internationale (FCI) group (model variables: FCI group + year), and clade [48,49] (Supplementary Materials). Six of the eight AKC groups accounted for in the prcd-PRA analysis were significant (Foundation Stock Service (consisting of Australian Stumpy Tail Cattle Dog, Bolognese, Bolonka Zwetna, German Spitz, Karelian Bear Dog, Lapponian Herder, and Swedish Lapphund), Herding, Non-sporting, Sporting, Toy, and Working) (Table S9). Additionally, in the FCI + Year analysis for prcd-PRA, all six of the represented FCI groups had significant *p*-values (Table S10). Of the clades present in the prcd-PRA dataset, six of fifteen clades were significant (Alpine, Unclustered1 (Finnish Lapphund), Poodle, Retriever, Nordic Spitz, and American Toy) (Table S11).

### 3.3. Trends in CEA

**Figure 8:**
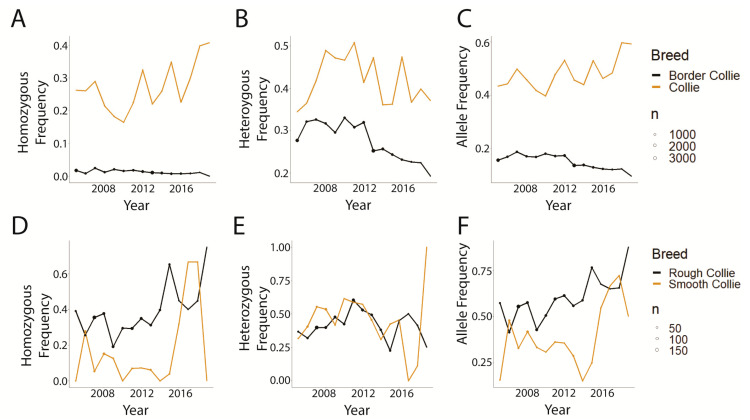
Genotype and allele frequencies in Border Collies and Collies tested for CEA (2005–2019). Point sizes indicate the number of dogs tested that year. (**A**–**C**) Border Collie and Collie. (**D**–**F**) Subsets of Collie, split into Rough Collie and Smooth Collie. (**A**,**D**) Homozygous variant genotype. (**B**,**E**) Heterozygous genotype. (**C**,**F**) Disease-associated variant allele.

Seven of the breeds/mixes tested for CEA were statistically significant and, therefore, determined to have a strong effect on the presence or absence of this disease and its associated genotypes (Hokkaido Dog, Collie, Boykin Spaniel, Silken Windhound, Longhaired Whippet, Shetland Sheepdog, and Border Collie) (Table 4, Table S12, Figure 8A–C). Of these, five breeds met the minimum of 50 dogs tested in at least half of the years; within those five breeds, the greatest log odds of possessing a homozygous-affected or heterozygous genotype was demonstrated in the Collie (regardless of regression model type). Additionally, the greatest frequency of the *NHEJ1* variant in a year (0.597 in 2018) was found in the Collie breed. The Shetland Sheepdog ranked second in all models, and both Collies and Shetland Sheepdogs were highly significant according to the logistic regression model (*p* < 2 × 10^−16^). (Table 4). The Border Collie ranked third in all models and was also significant (*p* = 0.00067) in the logistic regression model.

The Collie and Border Collie were selected for closer examination as the breeds with the greatest and least log odds, respectively, and for all models, among the breeds with significant *p*-values (Table 4). A strong decline in the frequency of homozygous-affected genotypes, heterozygous genotypes, and the disease-associated allele was demonstrated in Border Collies (Figure 8A–C) while an increase in homozygous-affected genotypes and the disease-associated allele was observed in Collies (Figure 8A,C). Collies that had a variety of designations were further separated into Smooth and Rough Collies subgroups (Figure 8D–F). In years with a larger sample size (until about 2014), all frequencies decreased in the Smooth Collies from 2005–2015 (Figure 8D–F), while the frequencies of the homozygous-affected genotype and the disease-associated allele increased in the Rough Collies (Figure 8D,F).

**Figure 9:**
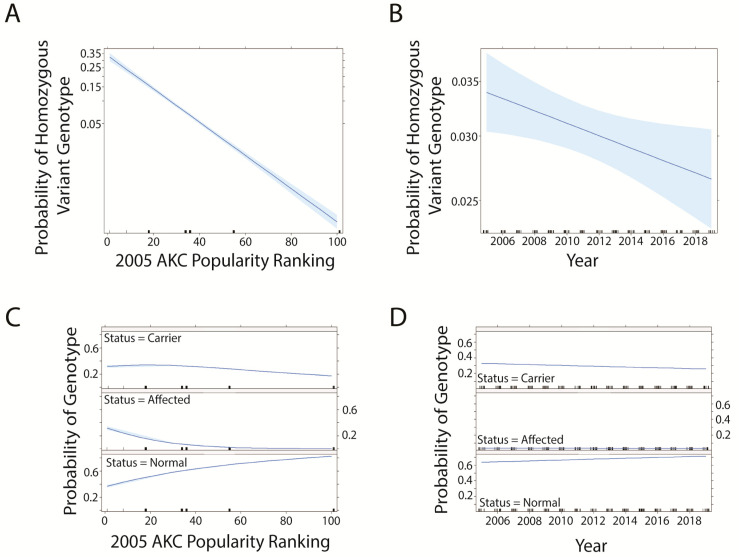
Effect of breed popularity according to 2005 AKC ranking by number of new registrations on the probability of CEA genotype in a model including breed rank and year. Blue shading indicates a 95% confidence interval. (**A**) Correlation between 2005 AKC popularity rank (with rank #1 = most popular breed) and probability of affected genotype (logistic regression model). (**B**) Correlation between year and probability of affected genotype (logistic regression model). (**C**) Correlation between 2005 AKC popularity rank and probability of affected, carrier, and normal genotypes (multinomial regression model). (**D**) Correlation between year and probability of affected, carrier, and normal genotypes (multinomial regression model).

The effect of breed popularity on a dog’s likelihood of being homozygous or heterozygous for the CEA-associated allele was determined using logistic and multinomial regression models including the variables of 2005 AKC rankings based on the number of new dogs registered for each breed (Table S13) and year. According to this ranking, the Labrador Retriever was the most popular breed included in the dataset (highest ranked, = rank #1) and the Nova Scotia Duck Tolling Retriever was the least popular breed (lowest ranked = rank #113). The probability of any dog having a homozygous-affected genotype or being a carrier for CEA decreased as the dog breed became less popular (Figure 9A,C). The progression of time was associated with decreasing probability of homozygous-affected and carrier genotypes (Figure 9B,D). Additional analyses using AKC rankings from 2019 (Table S14) displayed identical trends (Supplementary Materials, Figure S1).

**Figure 10:**
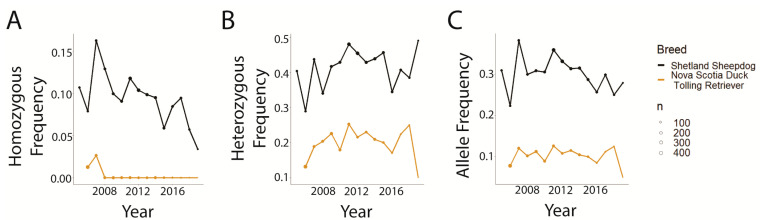
Global CEA genotype and allele frequencies for Shetland Sheepdog and Nova Scotia Duck Tolling Retriever. Point size indicates the number of dogs tested that year. (**A**) Homozygous variant genotype. (**B**) Heterozygous genotype. (**C**) Disease-associated variant allele.

A closer examination of two breeds, the Shetland Sheepdog and NSDTR, selected as breeds with very different 2005 popularity (rank of 18 for Shetland Sheepdog and rank of 113 for NSDTR), revealed that the popular breed (Shetland Sheepdog) had a higher frequency of homozygous-affected and heterozygous genotypes and disease-associated allele, at all time points, compared with NSDTR (Figure 10).

As with the prcd-PRA dataset, the CEA dataset was investigated to determine the effect of the AKC group, FCI group, and clade [48,49] (Supplementary Materials). In the AKC analysis, three of the five represented AKC groups were significant (Herding, Sporting, and Miscellaneous (Lancashire Heeler)) (Table S15). Conversely, none of the four FCI groups represented by the CEA dataset had a significant effect on log odds of a homozygous-affected or carrier genotype (Table S16). Similar to the FCI analysis, none of the represented clades were found to have a significant effect (Table S17).

## 4. Discussion

For both diseases, the spike in number of tests in the first year of data (CEA) and the peak in the first few years after test availability (prcd-PRA) indicate a strong surge of public interest in getting dogs tested for these diseases (Figure 1B). While prcd-PRA and CEA show different trends, both begin an overall decline in testing around 2013 with the number of samples continuing to decline as time progresses after that (Figure 1B). This is possibly due to the emergence of other available DNA laboratories testing for these diseases following the 2013 United States Supreme Court case, *Association for Molecular Pathology v Myriad Genetics*, which ruled that human genetic sequence is not patentable on the basis that it is naturally occurring [50]. Before this decision, OptiGen’s exclusively licensed patents on the tests for CEA and prcd-CEA resulted in the data presented here being representative of nearly global testing and trends. After 2013 and a later court case, *Genetic Veterinary Sciences, Inc. v. Canine EIC Genetics, LLC* [51], multiple canine genetic testing companies began offering prcd-PRA and CEA tests; thus, the declining numbers are likely best interpreted as testing being conducted by other providers, rather than an actual decrease in testing as both diseases were present worldwide (Figure 1A). Indeed, a 2022 Italian study of five dog breeds reported a 5.24% frequency of CEA *NHEJ1* variant homozygotes and a 31.45% carrier rate [41], while a 2019 Brazilian study reported a 25.5% prevalence of the prcd-PRA mutation in English Cocker Spaniels [45]. CEA and prcd-PRA were among the most common disease variants in a 2018 study (100,000 dogs) [13] and continue to be among the most prevalent disease variants in 2023 (over a million dogs) [12].

As with the global testing trends, all individual breed trends also exhibited a decline in test counts after 2013. Three common patterns emerged in testing trends amongst individual breeds: a right-tailed skew, unimodal normal distribution, and bimodal distribution (Figure S2). The driving forces behind breed-specific differences in test use and trends are likely tied to specific breed clubs and organizational guidelines regarding genetic testing; such policies also vary between countries within the same breed. For example, there are testing requirements for dogs in the United States and Canada (or other countries with case-by-case determination) to obtain a Canine Health Information Center (CHIC) number. CHIC makes health data publicly available to help inform breeding decisions and puppy purchases [52]. Interestingly, breeds with requirements for genetic testing for prcd-PRA or CEA designated as optional versus required in order to obtain a CHIC number [53] did not always correlate with those breeds receiving the most tests. Breeds with no or optional CHIC requirements were highly represented in the CEA dataset, such as Collies, Border Collies, and NSDTRs (Tables S4 and S18); conversely, it was the breeds requiring a prcd-PRA test that were generally the breeds with the greatest number of prcd-PRA tests in our dataset (Tables S4 and S18). Additionally, the uptake of genetic tests for prcd-PRA, but not CEA, may reflect breed size, as shown by the correlation, or lack thereof, respectively, between new AKC registrations in 2009 (akc.org/about/archive/digital-collections/ [accessed on 7 October 2023]; the most recent year with publicly available data for number of new registrations per breed) and the sample number of that breed tested in our dataset (Figure S3). Our results for both diseases contrast with previous research that determined that test uptake and breed population were negatively correlated [46]; this may be due to the fact that estimations of breed size in these data were taken from a single year of AKC registration data, the inclusion of a large number of dog breeds and breed mixes tested for each disease, the global perspective of this study, or a combination of these factors. Less comprehensive testing in more popular breeds may have also contributed to this negative correlation.

The size of this dataset enables the unique ability to closely gauge previous and current global trends for both diseases. The graphical analysis of genotypic and allelic frequencies over time demonstrates a clear decline for prcd-PRA, while the CEA trends remain more difficult to discern on visual inspection, with a less significant decline (Figure 1D,E) or even a potential increase (Figure 1C). While a chi-square goodness-of-fit analysis comparing the first and last data points showed a significant difference in genotype frequencies over time (Table 1), confirming the trend directions—particularly for CEA—ultimately required logistic regression and multinomial regression modeling. Models that included a dog’s breed, owner country, and year demonstrated via year coefficient that regardless of model, all are negative (Table 2), meaning that, even if breed and country remain constant, the likelihood of a dog having an affected or carrier genotype for either disease studied decreases with the progression of time. This can also be seen graphically (Figure 3 and Figure 4), and the impact of the year on log odds is statistically significant. Indeed, the year is significant in nearly all models which, taken together, indicates that the approximate global trends generally followed the hoped-for outcomes of a rapid elimination of affected dogs and a slow, more gradual elimination of the disease-associated variant allele.

Collies showed a significant departure from other breed trends in the CEA data. Collies not only had the greatest relative probability of affected genotype or carrier genotype for the CEA-associated variant (among breeds with *n* ≥ 50 in at least half the time points (Table 4)) but also clearly demonstrated graphically increasing frequencies of the affected genotype and CEA-associated genotype (Figure 8A–C). In the current CEA dataset, Collies could also be split into four groups: Rough Collies, Smooth Collies, FamCollies, and Collies with no identified variety. Comparing just the Rough and Smooth Collies revealed an interesting disparity. Rough Collies generally showed the more concerning trends of increasing homozygous variant genotypes and disease allele frequencies and Smooth Collies showed the opposite, with the exception of 2015 onward where the sample size dropped (accounting for a late spike) (Figure 8D–F). One potential driving force is that breeders may be using ophthalmic CEA phenotype severity to judge breeding decisions as opposed to the CEA-associated variant genotype, thereby reducing the overall severity of affected dogs (with fewer experiencing coloboma or retinal detachment) regardless of the presence of disease-associated genotype. However, 2021 statistics from the American College of Veterinary Medicine (https://ofa.org/diseases/eye-disease/blue-book/ [accessed on 7 October 2023]) reveal that the frequency of coloboma has increased within the Collie population alongside the less-severe chorioretinal hypoplasia. Another potential factor responsible for the observed trends is breeders subscribing to a long-held belief within the Collie breeder community; this belief is that the *NHEJ1* mutation is strongly associated with the desirable “almond eye” shape, explaining the high prevalence of CEA among show-quality Collies (Robette Johns, President of the Collie Health Foundation, personal communication). To date, there is no scientific literature supporting this proposed relationship. However, since CEA was typically considered a “mild” phenotype (not counting colobomas), and the breeders placed higher priorities elsewhere, CEA genotypes may not have weighed as heavily in mating decisions (Robette Johns, President of the Collie Health Foundation, personal communication).

The variable of country was owner-reported and represented the year in which the tests were submitted. As such, the countries analyzed in this study may not accurately reflect the current geopolitical relations and borders. Furthermore, a caveat to country (and breed) information is that it was owner-entered and, therefore, subject to possible entry error. This is demonstrated by looking into the single sample from Bouvet Island, which upon further investigation, likely originated from South Carolina, USA, although there is no way to definitively verify its origin. Such errors likely do not have a large overall effect, and in most cases, are nearly impossible to detect. Logistic and multinomial regression models for both diseases indicated that no country had a statistically significant effect on log odds of affected or carrier genotype status (Tables S19 and S20). Although the majority of genotype data stemmed from North America and Europe (Figure 1A), all seven continents are represented among the samples, lending a more global picture. Using LRM and MRM on a model including the variables of continent and year, only Africa was statistically significant in the prcd-PRA dataset (Table S21) and no continents were significant in the CEA analysis (Table S22). Additional analysis should be carried out with a larger prcd-PRA test sample size from Africa, representing more African countries, in order to determine if this is an artifact of the small African cohort in the current study. Taken together, this suggests that geographic location does not have a significant impact on log odds of a dog being homozygous or a carrier for either disease, neither due to geographic space/country nor to country- or continent-specific regulations regarding genetic testing.

Since it is very likely that trends observed in these data are the result of human behavior, as breeders selected which dogs to undergo testing prior to breeding, the influence of a dog breed’s popularity was also considered. Here, breed ranking was used as a proxy for popularity, according to the number of new dogs registered to the AKC, in the first year of data being analyzed. While using AKC registrations does not necessarily reflect the actual global popularity of breeds, these are the most concise, breed-specific, publicly available data, covering a large geographic area. Further, there is no guarantee that AKC-registered dogs solely reside in the United States. Interestingly, in the logistic and multinomial regression analyses, prcd-PRA and CEA exhibited opposing trends, where more popular breeds had lower probabilities of affected or carrier status for prcd-PRA (Figure 6) and less popular breeds had a lower likelihood of being homozygous-affected or carriers for CEA (Figure 9). Identical trends regarding the effect of rank on genotype and phenotype likelihoods when utilizing AKC rankings from 2019 (Table S14) indicate that this is a representative trend, and not an artifact of the year chosen (Supplementary Materials, Figure S1). One possible explanation is that prcd-PRA has a more severe impact on vision, prompting more rapid responses by breeders to reduce prevalence. In addition, less populous breeds with a smaller population size face the challenge of balancing the removal of the mutated allele from the breeding pool while maintaining genetic diversity within an already limited gene pool. Since the CEA trend was unexpected, it was further hypothesized that the overall trend might be strongly influenced by Collies, a highly ranked breed in 2005 (rank of 36); however, even after reanalysis without Collies included, the trend was unchanged (Supplementary Materials, Figure S4). It was considered that the significant impact of breed popularity on disease likelihood could be due to the amount of heterozygosity present in breeds of different popularity, which may impact the success of removing diseases from a population through the presence of genetic variance. To test this, the estimated median heterozygosity [12] for the breeds in this dataset was analyzed via regression models to determine the impact on the likelihood of disease status or genotype (Supplementary Materials). Regression results indicated that heterozygosity of a breed is negatively correlated with disease and genotype likelihood at a statistically significant level (*p* < 2 × 10^−16^ for both diseases) (Figure S5). Given that this trend does not differ in direction between the two diseases as breed rank analysis does, it is unlikely to be the direct cause of the conflicting trends further supported in correlation analyses with heterozygosity and breed popularity ranking (Figure S6). Recognizing that AKC popularity rankings do not necessarily reflect global popularity rankings, the breed popularity analysis should be repeated if global scale popularity data became available; this would provide a more global conclusion about the effect of breed popularity on the likelihood of a dog’s disease and genotype status for prcd-PRA and CEA.

Alternatively, breed genotype and allele frequency trends may relate more to a dog’s function, i.e., what a dog was bred to do, as captured by AKC and FCI groupings. While a large number of represented groups under the AKC and FCI were significant for prcd-PRA (Tables S9 and S10), only three of the AKC groups and none of the FCI groups were significant for CEA (Tables S15 and S16). This suggests that the purpose a breed was originally developed for does have some effect on how genotype and allele frequencies are trending for CEA and prcd-PRA. This could be due to human activity; for example, breeders of herding or working dogs adopt and act on genetic testing differently compared with breeders of retrieving dogs, or it could be due to genetic differences between dogs of different groups. For example, we considered that were *PRCD* or *NHEJ1* positioned on a chromosome close to a breed-linked behavior (herding, pointing, and hunting) locus with strong influence, selection for those behaviors might affect the prevalence of prcd-PRA and CEA-associated variants due to linkage disequilibrium. However, the genomic locations of seven herding-related genes (*MC2R*, *THOC1*, *ASIC2*, *MSRB3*, *LLPH*, *RFX8*, and *CHL1*) [54,55], three hunting genes (*LRRTM4*, *JAK2*, and *MEIS1*) [54], and one gene associated with pointing (*CNIH1*) [55] were compared with the locations of *PRCD* and *NHEJ1* according to CanFam3.1, and only *ASIC2* was on the same chromosome (chromosome 9) as *PRCD.* None shared a chromosome with *NHEJ1*. On CFA9, *ASIC2* and *PRCD* are separated by approximately 34 million base pairs. Using CanFam3.1 positions and an estimated sex average rate of recombination of 0.91 cM/Mb on CFA9 [56], the two genes were determined to be approximately 31.75 cM apart, suggesting moderate linkage with a 31.75% chance of recombination between the two during each meiosis event; therefore, they are certainly not in tight linkage disequilibrium.

Further investigation of genetic relationships and how they may impact prcd-PRA and CEA breed trends was undertaken using a model to investigate the impact of genetic clade [48,49]. While none of the represented clades were significant in the CEA set, half of the ones in the prcd-PRA dataset were (Tables S17 and S11), suggesting that some of the genotype probability varying by AKC and FCI group may be due to the genetic relatedness of included breeds. However, it is important to note that, while CEA is significant for the Herding group in the AKC group analysis (Table S15), in the genetic clade analysis, the UK Rural clade (which includes much of the herding group such as Collies and Shetland Sheepdogs) is not significant (Table S17). This suggests that while the genetic relationship may have some effect, it is not solely responsible for the trends observed in AKC or FCI group analysis on breed function; this may be due to the fact that the breeds involved in the formation of AKC and FCI groups were not necessarily closely genetically related.

Overall, this study demonstrates that commercial genetic testing for prcd-PRA and CEA is being used to inform breeding decisions to improve canine health, as evidenced by the progression of time being associated with significantly decreasing log odds of affected and carrier genotypes (Table 2). While both diseases show this encouraging trend, they do not behave identically, as prcd-PRA has a more significant decline in genotype probability (Table 1 and Table 2). This difference is also observed when comparing the same dog breeds tested across both diseases (Figure 2). Indeed, the genotype and allele frequencies are either lower for prcd-PRA (Figure 2A–C) or declining at a greater rate (Figure 2D–F) compared with CEA. While this is undoubtedly due to public interest in removing the blinding prcd-PRA disease from breed populations, it could be influenced by less accessible education regarding CEA risk or a different application approach for CEA by breeders. Future research could begin to tease out this answer by comparing a larger number of breeds tested for both diseases and observing if the different patterns remain despite different breed backgrounds. Further investigation into specific breeding practices adopted in distinct breed populations may explain why the two diseases are demonstrating different trends.

This study is unique in terms of the large sample size and lengthy continuous time period; however, there are limitations to its scope and conclusions. First, while these data include samples from all seven continents, the majority represent North America and Europe. Therefore, in countries and continents with smaller sample sizes, these analyses are unlikely to accurately reflect the full extent of genotype and allele frequency trends. Moreover, in this dataset, as mentioned above, country and dog breed or breed mix were owner-reported and, therefore, subject to human data entry error. Also, while likely to reflect the location an owner was living at the time of test submission, owner-reported data do not guarantee the country where the dog was born or where the dog’s bloodlines originated. And, like all large datasets, the size of this study also potentially allows for some insubstantial differences to be statistically significant, so some caution may need to be considered.

An additional limitation of this study is the lack of clinical information. The present study exclusively presents genetic testing results, and no information is available on any dog’s diagnosis, either before or after test submission. Although there is variation in the rate of retinal degeneration, dogs of any breed with two copies of the prcd-PRA variant almost invariably go blind as they age. Conversely, because CEA has a wide array of phenotype severity, including normal vision, no firm conclusions about disease status or clinical signs can be drawn about dogs homozygous for the *NHEJ1* deletion. In addition, some level of sampling bias likely affects these data, since a dog’s clinical disease status can influence genetic testing decisions. For example, dogs diagnosed with either disease may then undergo genetic testing as confirmation. Sampling bias also results when dogs designated as “parentage clear” (due to both parents testing homozygous clear) do not undergo testing. Therefore, affected genotype, carrier genotype, and variant allele frequencies in this study may be greater than the true frequencies. This could be rectified via a combined genetic and hereditary status approach [46], which was not possible with the present dataset and likely contributed to the conflicting prcd-PRA allele frequencies observed within this study.

Finally, it must be pointed out that while these trends are likely the outcome of direct action by breeders, it is also possible that they are the result of genetic drift and, therefore, simply artifacts of breeding decisions made with respect to other prioritized traits (e.g., conformation or performance). Such decision paradigms are undoubtedly influenced by the severity of the disease, with PRA likely motivating more direct action compared with CEA, but are also influenced by each breed community’s approaches and rules regarding genetic testing. While the trends identified in this study are highly encouraging, the remaining presence of homozygous-affected genotypes for both diseases in the final year of study (and supported by other work [12]) shows that continued use of genetic testing is needed to further improve genetic health. This is particularly true for prcd-PRA (10 affected of 1149 tested in 2019; 9.8% disease allele frequency), where these dogs almost certainly have or will experience retinal degeneration. It is possible these affected dogs are the result of crosses between unknown carriers; however, in the absence of compulsory parentage verification, incorrect puppy registration can and likely does occur (either due to human error or intentional misrepresentation), leading to the inadvertent creation of affected puppies. This underscores the value of genetic testing in combination with parentage verification. To continue improving, it is important that breeders utilize genetic testing and make careful, deliberate breeding choices. Ideally, no more affected puppies would be produced. However, immediately removing all carrier dogs from a breeding pool is discouraged, as it can drastically reduce genetic diversity within a breed, and then increase the prevalence of other deleterious mutations. Indeed, this occurred historically in the Portuguese Water Dog; removing GM1-gangliosidosis carriers in the 1960s–1970s led to an increase in the prevalence of prcd-PRA breed-wide [57]. Carrier dogs that are otherwise excellent can and should be maintained within the breeding pool, so long as they are only bred to guaranteed clear dogs, thereby ensuring that they produce no homozygous-affected puppies. Eventually, as this strategy progresses, the puppies selected to continue breeding will be clear of prcd-PRA or CEA, thus improving overall health while still maintaining genetic diversity. This proposed strategy could explain the peak and consistent frequency of prcd-PRA heterozygotes in Sweden (Figure 3B) in conjunction with the declining frequency of affected dogs and the disease-causing allele (Figure 3A,C). The combination of these findings suggests that breeders in Sweden are taking steps to reduce the prevalence of dogs that will go blind, as well as the overall prevalence of the causal mutation, while still maintaining carriers and, therefore, overall genetic diversity. As this strategy continues, the carrier frequency would be expected to slowly decline overall; future work measuring these trends would definitively demonstrate if this is the case.

## 5. Conclusions

This study is a first-of-its-kind examination of how commercial genetic testing is used, and to what extent, to influence disease-associated genotype frequencies for two congenital ocular diseases, utilizing a global dataset with over 120,000 individual genetic test results. This study presents a continuous fifteen-year cohort of 67 breeds and breed mixes and 82 countries globally using commercial genetic testing results to reduce the frequency of affected and carrier dogs, as well as the overall disease-associated (alternate) allele frequency. Generally, the geographic distribution of tests did not have a significant impact on genotype probability, although breed, AKC group, FCI group, and breed popularity did. Prcd-PRA, the more severe of the two diseases, exhibited a more significantly decreasing frequency trend compared with CEA, which may indicate a stronger priority amongst breeders to remove prcd-PRA from the population. Considering that affected dogs were observed for both CEA and prcd-PRA in the 2019 data, it is important that genetic testing continues, with careful test-and-replace strategies in order to maintain genetic diversity because while progress has been made, there is still more work that needs to be carried out. Future work should continue this line of study, perhaps by pooling genetic testing data from many genetic testing providers over the same time period in order to gain a truly globally distributed dataset with a larger cohort of dogs for the two present and other different diseases; this would provide highly accurate global trends. Additionally, updated analyses including data from 2020 to the present would allow scrutiny of the most current trends. To aid in this strategy, lay public-accessible summary tables are available in the Supplemental Materials (Tables S23 and S24).

## Figures and Tables

**Table 1:** Chi-square goodness-of-fit test comparing genotype frequencies of the first and last years of data.

	X^2^	*p*-Value
prcd-PRA	698.0194	2.7 × 10^−152^
CEA	7.52339	0.023244

**Table 2:** Year coefficient describing the effect of year on log odds of affected or carrier genotype for prcd-PRA and CEA across the timeframe of the study.

	LRM for Affected Genotype (*p*-Value)	MRM for Genotype (SE)	MRM for Carrier Genotype (SE)
prcd-PRA	−1.252 × 10^−1^ (<2 × 10^−16^)	−0.04864 (1.07 × 10^−5^)	−0.06331 (5.54 × 10^−6^)
CEA	−2.46 × 10^−2^ (0.00834)	−0.03438 (1.62 × 10^−5^)	−0.01948 (9.95 × 10^−6^)

LRM = Logistic Regression Model, MRM = Multinomial Regression Model, SE = standard error.

**Table 3:** Relative rank of dog breeds ^1^ comparing prcd-PRA-affected to -unaffected status (LRM) and affected or carrier to clear status (MRM) according to model-generated measure of likelihood (log-odds ratio) from greatest likelihood to least.

Rank	LRM for Affected Genotype (*p*-Value *)	MRM for Affected Genotype (SE)	MRM for Carrier Genotype (SE)
1	Swedish Lapphund (**4.65 × 10^−5^**)	Swedish Lapphund (7.031 × 10^−4^)	Swedish Lapphund (4.300 × 10^−3^)
2	American Eskimo Dog (0.921564)	Entlebucher Mountain Dog (2.957 × 10^−4^)	American Eskimo Dog (3.057 × 10^−5^)
3	Entlebucher Mountain Dog (0.97598)	American Eskimo Dog (1.597 × 10^−6^)	Entlebucher Mountain Dog (1.865 × 10^−3^)
4	Australian Cattle Dog (0.955735)	Australian Cattle Dog (1.073 × 10^−3^)	Nova Scotia Duck Tolling Retriever (1.626 × 10^−2^)
5	English Cocker Spaniel (**0.006659**)	English Cocker Spaniel (2.299 × 10^−3^)	Australian Cattle Dog (6.342 × 10^−3^)
6	Spanish Water Dog (**0.000362**)	Nova Scotia Duck Tolling Retriever (6.288 × 10^−4^)	English Cocker Spaniel (2.005 × 10^−2^)
7	Finnish Lapphund (**4.75 × 10^−10^**)	Spanish Water Dog (2.990 × 10^−5^)	Labradoodle, Australian (1.363 × 10^−3^)
8	Nova Scotia Duck Tolling Retriever (**<2 × 10^−16^**)	Finnish Lapphund (7.442 × 10^−5^)	Dwarf Poodle (8.613 × 10^−4^)
9	Toy Poodle (**<2 × 10^−16^**)	Toy Poodle (1.058 × 10^−3^)	Finnish Lapphund (1.702 × 10^−3^)
10	Chesapeake Bay Retriever (**4.31 × 10^−8^**)	Chesapeake Bay Retriever (2.371 × 10^−5^)	Chesapeake Bay Retriever (6.065 × 10^−4^)
11	Labrador Retriever (**<2 × 10^−16^**)	Labrador Retriever (6.113 × 10^−4^)	Spanish Water Dog (6.533 × 10^−4^)
12	Miniature Poodle (**<2 × 10^−16^**)	Miniature Poodle (7.277 × 10^−4^)	Portuguese Water Dog (8.761 × 10^−3^)
13	Dwarf Poodle (**3.16 × 10^−6^**)	Dwarf Poodle (1.075 × 10^−5^)	Toy Poodle (2.562 × 10^−2^)
14	Portuguese Water Dog (**<2 × 10^−16^**)	Portuguese Water Dog (1.544 × 10^−4^)	Miniature Poodle (2.593 × 10^−2^)
15	Chinese Crested (**<2 × 10^−16^**)	Labradoodle, Australian (2.312 × 10^−5^)	Labrador Retriever (1.638 × 10^−2^)
16	Labradoodle, Australian (**4.71 × 10^−13^**)	Chinese Crested (1.439 × 10^−5^)	Golden Retriever (1.280 × 10^−3^)
17	Golden Retriever (**<2 × 10^−16^**)	Australian Shepherd (4.887 × 10^−6^)	Chinese Crested (5.514 × 10^−4^)
18	Australian Shepherd (**1.64 × 10^−9^**)	Golden Retriever (7.927 × 10^−6^)	Australian Shepherd (2.257 × 10^−4^)

LRM = logistic regression model; MRM = multinomial regression model; and SE = standard error. ^1^Minimum of 50 dogs tested in at least half of the time points. *Significant *p*-values are bolded.

**Table 4:** Relative rank of dog breeds ^1^ comparing CEA-affected to -unaffected status (LRM) and affected or carrier to clear status (MRM) according to a model-generated measure of likelihood (log-odds ratio) from greatest likelihood to least.

Rank	LRM for Affected Genotype (*p*-Value *)	MRM for Affected Genotype (SE)	MRM for Carrier Genotype (SE)
1	Collie (**<2 × 10^−16^**)	Collie (1.633 × 10^−2^)	Collie (2.571 × 10^−2^)
2	Shetland Sheepdog (**<2 × 10^−16^**)	Shetland Sheepdog (8.510 × 10^−3^)	Shetland Sheepdog (2.396 × 10^−2^)
3	Border Collie (**0.00067**)	Border Collie (8.850 × 10^−3^)	Border Collie (2.293 × 10^−2^)
4	Australian Shepherd (0.96233)	Australian Shepherd (5.506 × 10^−6^)	Nova Scotia Duck Tolling Retriever (3.209 × 10^−2^)
5	Nova Scotia Duck Tolling Retriever (0.38093)	Nova Scotia Duck Tolling Retriever (3.634 × 10^−4^)	Australian Shepherd (3.693 × 10^−5^)

LRM = logistic regression model; MRM = multinomial regression model; and SE = standard error. ^1^Minimum of 50 dogs tested in at least half of the time points. *Significant *p*-values are bolded.

## Data Availability

All raw data has been deposited in Dryad at the following URL: https://doi.org/10.5061/dryad.6djh9w17c.

## Supplementary Materials

The following supporting information can be downloaded at: https://www.mdpi.com/article/10.3390/genes14112093/s1, Figure S1: Effect of breed popularity according to AKC ranking by number of new 2019 registrations (actual counts not provided) and the probability of prcd-PRA and CEA genotypes in the model including breed rank and year; Figure S2: Examples of breed-specific test count distribution; Figure S3: Correlation between breed size according to number of 2009 new AKC registered dogs and test uptake as LOESS curve; Figure S4: Effect of breed popularity according to AKC ranking by number of new 2005 registrations on probability of CEA genotypes in model including breed rank and year, without Collies included; Figure S5: Effect of median breed percentage heterozygosity on probability of disease genotypes in models including median breed percentage heterozygosity and year; Figure S6: Correlation between breed popularity percentage median breed heterozygosity as LOESS curve; Table S1: Summary of dog breeds with presence of prcd-PRA causal *PRCD* variant; Table S2: Summary of dog breeds with presence of CEA-associated *NHEJ1* deletion; Table S3: Number of tests for prcd-PRA and CEA according to owner-identified country; Table S4: Number of tests for prcd-PRA and CEA according to owner-identified breed or breed mix; Table S5: Breeds and breed mixes emphasized in paper that met inclusion criteria of at least 50 dogs tested per year for at least eight years; Table S6: Breeds and breed mixes in dataset with disease-associated allele; Table S7: Relative rank of dog breeds and breed mixes comparing prcd-PRA-affected to -unaffected status (LRM) and affected or carrier to clear status (MRM) according to model-generated measure of likelihood (log-odds ratio) from greatest likelihood to least in models with global dataset including breed, country, and year variables; Table S8: Breeds in prcd-PRA dataset ranked by number of new dogs registered to AKC in 2004; Table S9: Relative rank of AKC groups comparing prcd-PRA-affected to -unaffected status (LRM) and affected or carrier to clear status (MRM) according to model-generated measure of likelihood (log-odds ratio) from greatest likelihood to least in models with global dataset including AKC group and year variables; Table S10: Relative rank of FCI groups comparing prcd-PRA-affected to -unaffected status (LRM) and affected or carrier status (MRM) according to model-generated measure of likelihood (log-odds ratio) from greatest likelihood to least according to models with global dataset including FCI group and year variables; Table S11: Relative rank of clades comparing prcd-PRA-affected to -unaffected status (LRM) and affected or carrier to clear status (MRM) according to model-generated measure of likelihood (log-odds ratio) from greatest likelihood to least according to models with global dataset including clade and year variables; Table S12: Relative rank of all breeds and breed mixes comparing CEA-affected to -unaffected status (LRM) and affected or carrier to clear status (MRM) according to model-generated measure of likelihood (log-odds ratio) from greatest likelihood to least according to models with global dataset including breed, country, and year variables; Table S13: Breeds in CEA dataset ranked by number of new dogs registered to AKC in 2005; Table S14: Breeds in prcd-PRA and CEA dataset ranked by number of new dogs registered to AKC in 2019; Table S15: Relative rank of AKC groups comparing CEA-affected to -unaffected status (LRM) and affected or carrier to clear status (MRM) according to model-generated measure of likelihood (log-odds ratio) from greatest likelihood to least according to models with global dataset including AKC group and year variables; Table S16: Relative rank of FCI groups comparing CEA-affected to -unaffected status (LRM) and affected or carrier to clear status (MRM) according to model-generated measure of likelihood (log-odds ratio) from greatest likelihood to least according to models with global dataset including FCI group and year variables; Table S17: Relative rank of clades comparing CEA-affected to -unaffected status (LRM) and affected or carrier to clear status (MRM) according to model-generated measure of likelihood (log-odds ratio) from greatest likelihood to least according to models with global dataset including clade and year variables; Table S18: CHIC* enforcement of genetic testing for prcd-PRA and CEA in breeds included in dataset; Table S19: Relative rank of countries comparing prcd-PRA-affected to -unaffected status (LRM) and affected or carrier to clear status (MRM) according to model-generated measure of likelihood (log-odds ratio) from greatest likelihood to least according to models with global dataset including breed, country, and year variables; Table S20: Relative rank of countries comparing CEA-affected to -unaffected status (LRM) and affected or carrier to clear status (MRM) according to model-generated measure of likelihood (log-odds ratio) from greatest likelihood to least according to models with global dataset including breed, country, and year variables; Table S21: Relative rank of continents comparing prcd-PRA-affected to -unaffected status (LRM) and affected or carrier to clear status (MRM) according to model-generated measure of likelihood (log-odds ratio) from greatest likelihood to least according to models with global dataset including breed, continent, and year variable; Table S22: Relative rank of continents comparing CEA-affected to -unaffected status (LRM) and affected or carrier to clear status (MRM) according to model-generated measure of likelihood (log-odds ratio) from greatest likelihood to least according to models with global dataset including breed, continent, and year variables; Table S23: Lay public summary table of overall prcd-PRA genotype and allele frequencies and allele frequency trend direction. This does not reflect the range of sample size across time or the magnitude of the trend; Table S24: Lay public summary table of overall CEA genotype and allele frequencies and allele frequency trend direction. This does not reflect the range of sample size across time or the magnitude of the trend.
