# Supplementary material for: Global Frequency Analyses of Canine Progressive Rod-Cone Degeneration–Progressive Retinal Atrophy and Collie Eye Anomaly Using Commercial Genetic Testing Data
Source: Genes (Basel). 2023 Nov 17;14(11):2093. doi: 10.3390/genes14112093 (PMC10671078; doi:10.3390/genes14112093)
Supplement: Supplementary file 1 [file genes-14-02093-s001.zip › Final_SupplementalMethods_10_6_23_UpdatedReferences.pdf]

**Supplemental Methods**

For both diseases, each sample was assigned a continent (newworldencyclopeida.org), AKC group (akc.org), FCI group (fci.be/en/), and clade [48,49]. Next, logistic and multinomial regression models weighted by count were performed on models including: 1) breed + continent + year, 2) AKC group + year, 3) FCI group + year, and 4) clade + year. The variables of continent, AKC group, FCI group, and clade are categorical variables, and year of test was continuous. For breeds not included in clade reference studies, but for which internal databases contained single nucleotide polymorphism (SNP) data (Boykin Spaniel, Chesapeake Bay Retriever, Entlebucher Mountain Dog, Finnish Lapphund, Lapponian Herder, Miniature American Shepherd, Miniature Australian Shepherd, Spanish Water Dog), a phylogenetic analysis using internal SNP data determined which clade each was most related to. Otherwise, if a sample could not be included in an AKC group, FCI group, or clade the sample was removed from that model. After completion of modeling, all variables were ordered from greatest to least relative log-odds of having a homozygous affected or heterozygous genotype.

To determine an association between breed population size and uptake of genetic tests for CEA and prcd-PRA, the number of new AKC 2009 registrations (the most recent publicly-available data with breed-specific new registration counts) was used to estimate overall size of breed; this was plotted against the number of tests of that breed. Associations are shown with a LOESS curve and a 95% confidence interval. Finally, to further evaluate the effect of Collie trends in the overall influence of breed popularity on CEA genotype likelihood, breed rank according to 2005 AKC registrations together with year were used to create LRM and MRM models with global data not including Collies.

Median percentage heterozygosity data for each breed in this study was obtained from a recent paper that studied more than one million dogs [12]. Logistic and regression models using the variables of % median heterozygosity + year were run to determine the impact of breed heterozygosity on likelihood of disease state and genotypes. Additionally, median percentage heterozygosity was plotted against breed popularity to determine association with a LOESS curve and 95% confidence interval.
