# Supplementary material for: Global Frequency Analyses of Canine Progressive Rod-Cone Degeneration–Progressive Retinal Atrophy and Collie Eye Anomaly Using Commercial Genetic Testing Data
Source: Genes (Basel). 2023 Nov 17;14(11):2093. doi: 10.3390/genes14112093 (PMC10671078; doi:10.3390/genes14112093)
Supplement: Supplementary file 1 [file genes-14-02093-s001.zip › Final_Supplemental_FiguresTables_10_6_2023_Updated References.pdf]

Supplementary Figures and Tables

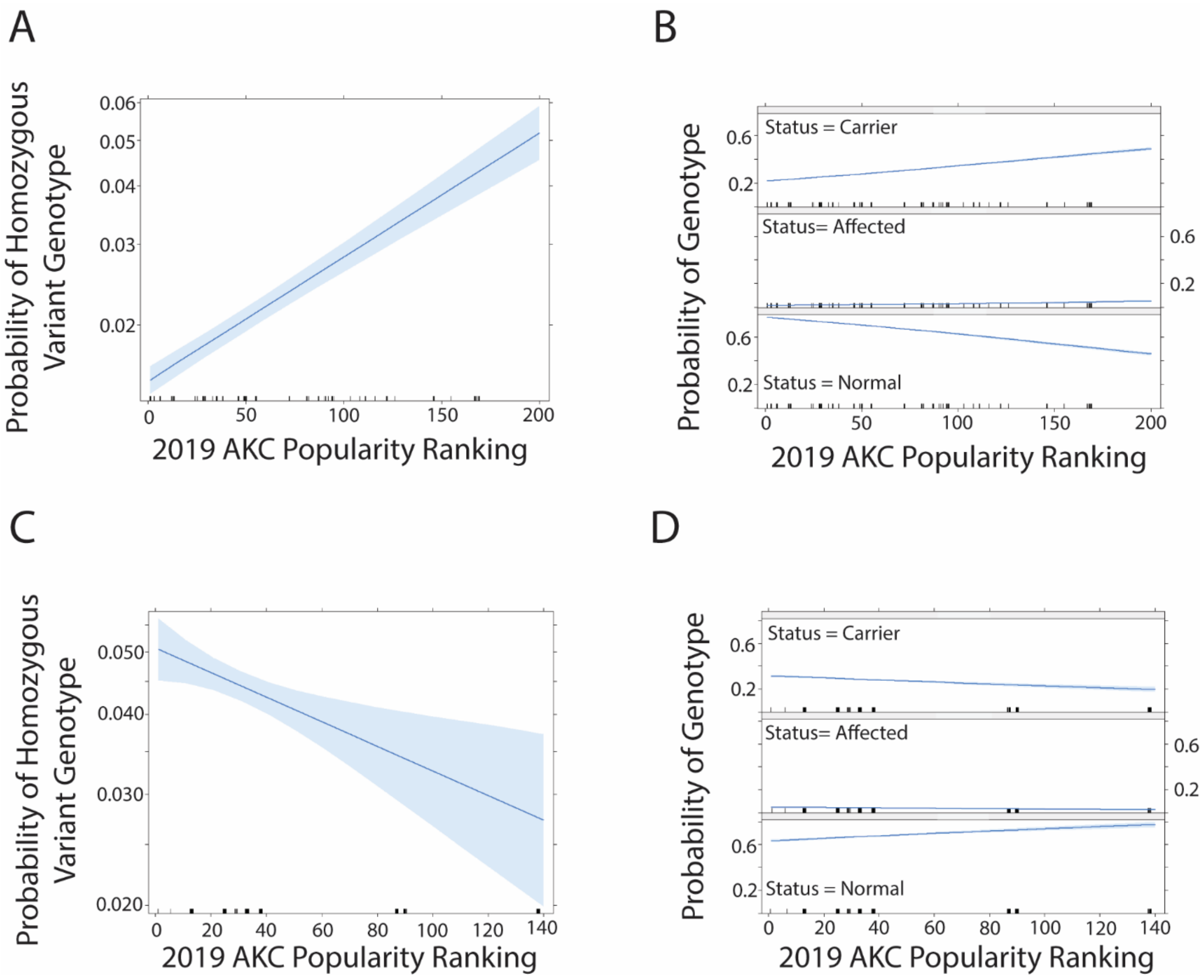

Figure S1. Effect of breed popularity according to AKC ranking by number of new 2019 registrations (actual counts not provided) the probability of prcd-PRA and CEA genotypes in model including breed rank and year. Blue shading indicates 95% confidence interval. (A) Effect of breed rank in LRM model for prcd-PRA, (B) effect of breed rank in MRM model for prcd-PRA, (C) effect of breed rank in LRM model for CEA, (D) effect of breed rank in MRM model for CEA

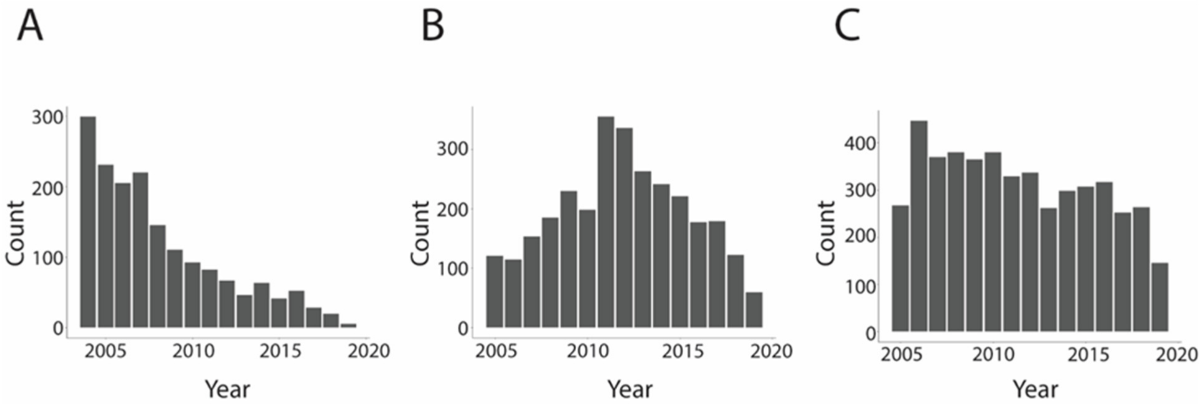

Figure S2. Examples of breed-specific test count distribution. (A) American Eskimo Dog prcd-PRA tests, (B) Shetland Sheepdog CEA tests, (C) Portuguese Water Dog prcd-PRA tests

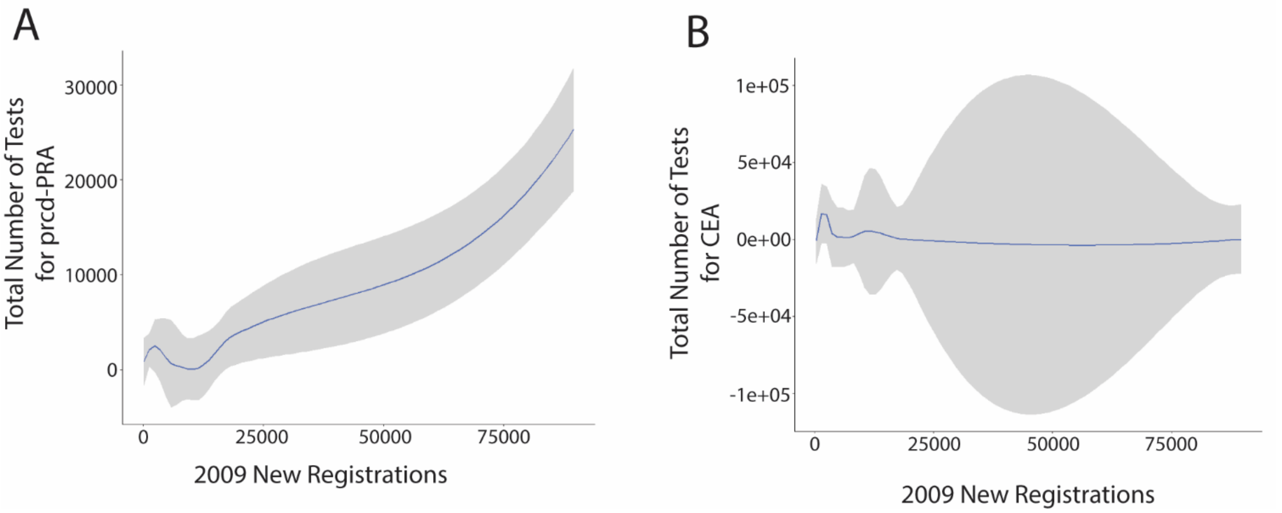

Figure S3. Correlation between breed size according to number of 2009 new AKC registered dogs and test uptake as LOESS curve. Gray shading indicates 95% confidence interval. (A) prcd-PRA, (B) CEA

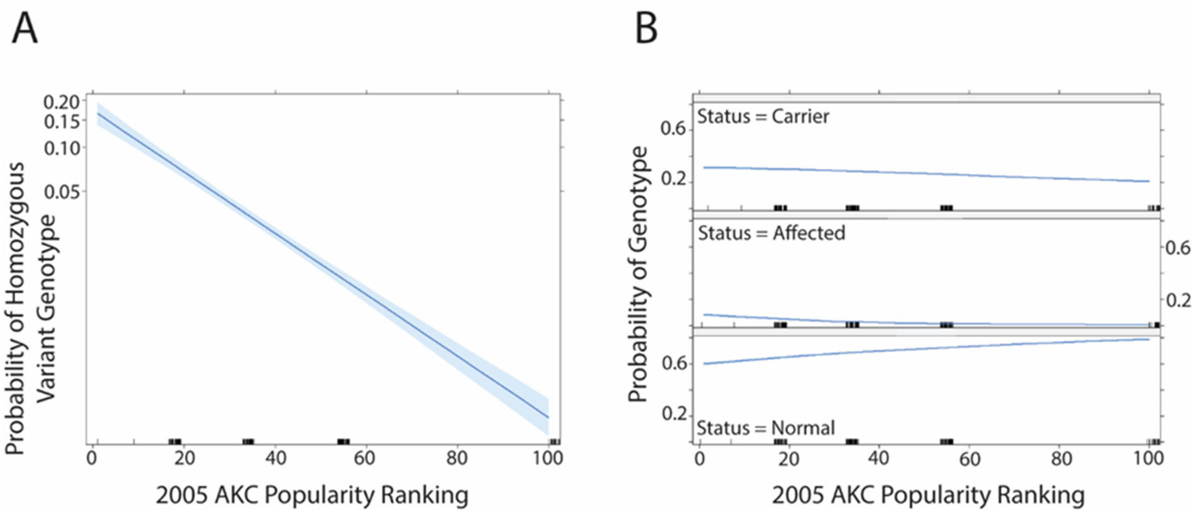

Figure S4. Effect of breed popularity according to AKC ranking by number of new 2005 registrations on probability of CEA genotypes in model including breed rank and year, without Collies included. Blue shading indicates 95% confidence interval. (A) Effect of breed rank in LRM model, (B) effect of breed rank in MRM model

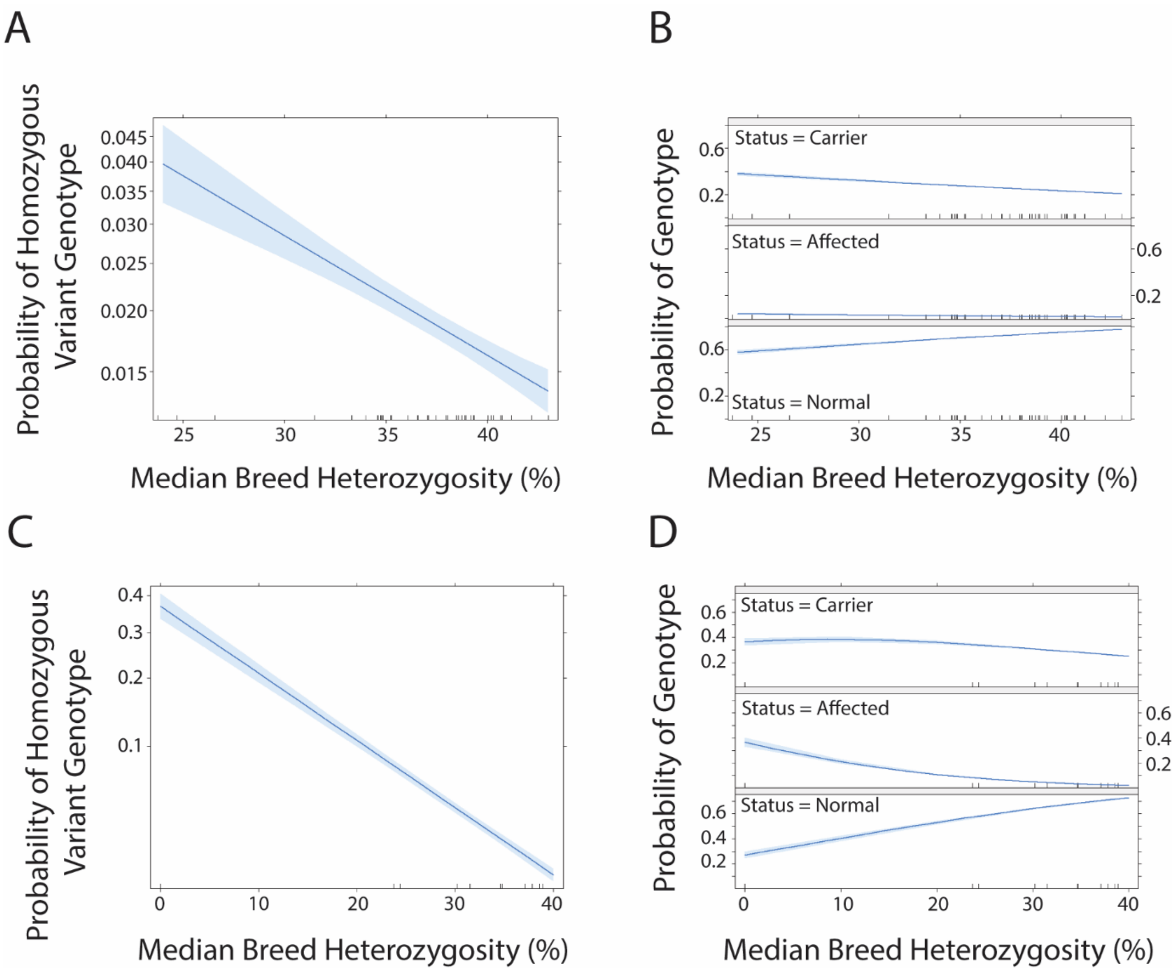

Figure S5. Effect of median breed percentage heterozygosity on probability of disease genotypes in models including median breed percentage heterozygosity and year. Blue shading indicates 95% confidence interval. (A) Effect of heterozygosity on prcd-PRA in LRM model, (B) effect of heterozygosity on prcd-PRA in MRM model, (C) effect of heterozygosity on CEA in LRM model, (D) effect of heterozygosity on CEA in MRM model

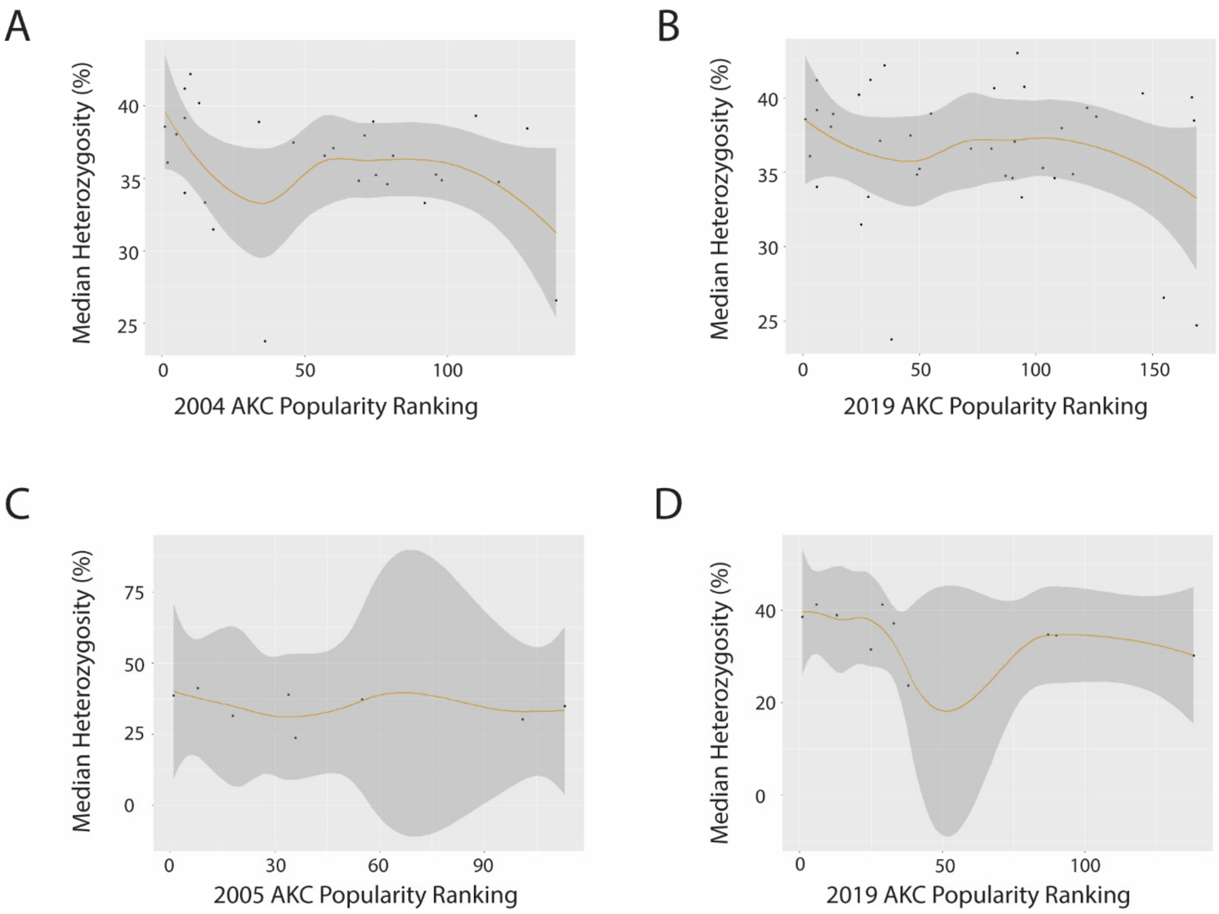

Figure S6. Correlation between breed popularity percentage median breed heterozygosity as LOESS curve. Gray shading indicates 95% confidence interval. (A) prcd-PRA with 2004 breed rankings, (B) prcd-PRA with 2019 breed rankings,(C) CEA with 2004 breed rankings, (D) CEA with 2019 breed rankings

Table S1. Summary of dog breeds with presence of prcd-PRA causal *PRCD* variant

**Causal *PRCD* variant present in current dataset**	**Breed**	**Literature Reference**	**Online Reference[1]**
√	American Cocker Spaniel	[10]	
√	American Eskimo Dog	[11]	
	American Hairless Terrier		Pawprint[2], MyBreedData[6]
√	Australian Cattle Dog	[11]	
	Australian Cobberdog		UCDavis[3]
	Australian Koolie		Pawprint[2]
	Australian Labradoodle		UCDavis[3], MyBreedData[6]
√	Australian Shepherd	[11]	
√	Australian Shepherd, Miniature	[11]	Pawprint[2]
√	Australian Shepherd, Toy		Pawprint[2]
√	Australian Stumpy Tail Cattle Dog	[11]	
√	Barbet		Pawprint[2], MyBreedData[6]
	Berger d'Auvergne		MyBreedData[6]
	Bichon Frise	[12]	
	Biewer Terrier	[13]	
	Black Russian Terrier	[13]	
√	Bolognese	[13]	
√	Bolonka Zwetna	[15]	
	Carolina Dog		Pawprint[2]
	Cavalier King Charles Spaniel		Pawprint[2]
√	Chesapeake Bay Retriever	[10]	
	Chihuahua	[14]	
√	Chinese Crested	[10]	
√	Coton de Tulear	[13]	
	Dalmatian	[12]	
√	Dwarf Poodle	[11]	
√	English Cocker Spaniel	[10]	
	English Shepherd	[15]	
√	Entlebucher Mountain Dog	[11]	
	Field Spaniel	[13]	
√	Finnish Lapphund	[11]	
	German Spitz		UCDavis[3]
√	Giant Schnauzer	[15]	
√	Golden Retriever	[11]	
	Havanese		MyBreedData[6]
	Jack Russell Terrier	[13]	
	Japanese Chin	[13]	
	Kai Ken	[12]	
√	Karelian Bear Dog	[11]	
	Kerry Blue Terrier		MyBreedData[6]
	Koolie		Pawprint[2]
√	Kuvasz	[11]	
√	Labrador Retriever	[10]	
	Lagotto Romagnolo	[13]	
	Lancashire Heeler	[13]	
√	Lapponian Herder	[11]	
	Maltese		MyBreedData[6]
	Manchester Terrier – Toy	[13]	
	Markiesje	[11]	
	Mi-Ki	[13]	

√	Miniature American Shepherd		OFA[4]
√	Miniature Poodle	[10]	
√	Moyen/Klein Poodle	[11]	
	Norrbottenspitz	[13]	
√	Norwegian Elkhound	[11]	
√	Nova Scotia Duck Tolling Retriever	[10]	
	Plott	[13]	
	Pomeranian	[13]	
√	Poodle	[15]	
	Portuguese Podengo – Miniature	[13]	
√	Portuguese Water dog	[10]	
	Puli	[13]	
	Pyrenean Sheepdog		MyBreedData[6]
	Rat Terrier		Pawprint[2], OFA[4], MyBreedData[6]
	Russian-European Laika	[13]	
	Sarplaninac		MyBreedData[6]
√	Schipperke	[10]	
	Schnauzer (Miniature)	[12]	
	Serbian Hound	[13]	
√	Silky Terrier		OMIA[5], Pawprint[2], OFA[4], MyBreedData[6]
√	Spanish Water Dog	[11]	
	Swedish Jamthund		UCDavis[3]
√	Swedish Lapphund	[11]	
	Tibetan Terrier	[13]	
	Toy Fox Terrier		Pawprint[2]
√	Toy Poodle	[10]	
	Xolointzcuintle	[13]	
√	Yorkshire Terrier	[11]	

[1]Online references included for breeds for which the variant was not identified in published scientific literature

[2] https://www.pawprintgenetics.com/products/tests/details/89/ (Accessed 2/13/2023)

[3] https://vgl.ucdavis.edu/test/pra-prcd (Accessed 2/22/2023)

[4]https://ofa.org/all-dna-tests/?TEST=PROGRESSIVE%20RETINAL%20ATROPHY (Accessed 2/13/2023)

[5]https://omia.org/OMIA000218/9615/ (Accessed 2/22/2023)

[6]https://mybreeddata.com/ (accessed 10/2/2023)

Table S2. Summary of dog breeds with presence of CEA-associated *NHEJ1* deletion

***NHEJ1* deletion present in current dataset**	**Breed**	**Literature Reference**	**Online Reference[1]**
	Alaskan Sled Dog	[33]	
	Anatolian Shepherd Dog	[12]	
	Australian Kelpie	[13]	
√	Australian Shepherd	[34]	
√	Australian Shepherd, Miniature		OFA[2], Pawprint[3], MyBreedData[5]
√	Bearded Collie		OFA[2], Pawprint[3], MyBreedData[5]
	Belgian Sheepdog		MyBreedData[5]
√	Berger d'Auvergne		MyBreedData[5], https://www.chienbergerdauvergne.org/la-sante/
√	Border Collie	[34]	
√	Boykin Spaniel	[29]	
	Chinook	[13]	
√	English Shepherd		Pawprint[3]
√	Hokkaido Dog	[35]	
	Jack Russell Terrier	[13]	
	Koolie	[13]	
	Lacy Dog	[12]	
√	Lancashire Heeler	[29]	
	Lapponian Herder	[13]	
√	Longhaired Whippet	[29]	
	Maremma Sheepdog	[12]	
	McNab Dog	[12]	
√	Miniature American Shepherd		OFA[2], Pawprint[3]
√	Nova Scotia Duck Tolling Retriever	[29]	
	Old English Sheepdog		OMIA[4]
	Old-Time Scotch Collie		Pawprint[3]
	Ovelheiro Gaúcho		MyBreedData[5]
	Parson Russell Terrier	[13]	
	Polar Husky	[33]	
√	Rough Collie	[34]	
	Scottish Collie		Pawprint[3]
√	Shetland Sheepdog	[34]	
	Siberian Husky	[33]	
√	Silken Windhound		OMIA[4], OFA[2], Pawprint[3]
	Smithfield Collie		OMIA[4]
√	Smooth Collie	[34]	
	Tamaskan Dog	[13]	
	Welsh Sheepdog		MyBreedData[5]
	White Swiss Shepherd		MyBreedData[5]

[1]Online references included for breeds for which the variant was not identified in published scientific literature

[2]https://ofa.org/all-dna-tests/?TEST=PROGRESSIVE%20RETINAL%20ATROPHY (Accessed 2/13/2023)

[3] https://www.pawprintgenetics.com/products/tests/details/92/ (Accessed 2/13/2023)

[4]https://omia.org/OMIA000218/9615/ (Accessed 2/22/2023)

[5]https://mybreeddata.com/ (accessed 10/2/2023)

Table S3. Number of tests for prcd-PRA and CEA according to owner-identified country

**Country**	prcd-PRA	CEA
Andorra	1	
Argentina	11	26
Australia	649	593
Austria	536	842
Belarus		1
Belgium	630	920
Bermuda	2	
Bosnia and Herzegovina	1	
Bouvet Island		1
Brazil	301	509
Bulgaria	8	1
Canada	4543	1085
Chile	4	3
China	1	
Colombia	1	5
Costa Rica	4	
Croatia	13	34
Cyprus	22	
Czech Republic	335	444
Denmark	2392	613
Dominican Republic	1	
Estonia	91	4
Faroe Islands		83
Finland	3786	1041
France	3111	2565
French Polynesia	1	
Gabon	3	
Germany	5227	3793
Gibraltar	2	
Greece	15	3
Greenland	1	
Guadeloupe	2	
Hungary	192	123
Iceland	277	48
Ireland	178	842
Israel	34	
Italy	1014	1095
Japan	125	42
Kuwait		1
Latvia	18	16

Liechtenstein	2	3
Lithuania	14	2
Luxembourg	31	44
Macau	1	
Macedonia	2	
Malta	1	1
Mexico	9	3
Netherlands	2408	1050
Netherlands Antilles	1	1
New Caledonia	6	3
New Zealand	113	64
Norfolk Island		1
Norway	2484	548
Oman	1	
Panama	1	
Paraguay	2	
Peru		9
Poland	297	227
Portugal	66	27
Puerto Rico	2	
Qatar	1	
Reunion	19	16
Romania	20	2
Russia	268	55
Serbia-Montenegro	13	4
Singapore	1	1
Slovakia	70	43
Slovenia	33	48
South Africa	166	20
South Korea	5	
Spain	260	246
Sweden	7501	1077
Switzerland	936	1175
Taiwan	2	
Thailand	2	
Trinidad and Tobago	1	
Turkey		9
Ukraine	17	1
United Kingdom	13254	7198
Uruguay	14	1
USA	35111	7221
Zimbabwe		1

Table S4. Number of tests for prcd-PRA and CEA according to owner-identified breed or breed mix

**Breed/Mix**	prcd-PRA	CEA
American Eskimo Dog	1704	
American Hairless (Rat) Terrier	1	
Australian Cattle Dog	3798	
Australian Cobberdog	1	
Australian Shepherd	1636	3234
Australian Shepherd, Miniature	474	392
Australian Shepherd, Toy	76	26
Australian Stumpy Tail Cattle Dog	59	
Barbet	21	
Bearded Collie		355
Berger d'Auvergne		9
Black Russian Terrier	2	
Bolognese	36	
Bolonka Zwetna	53	
Border Collie	1	20588
Boykin Spaniel	1	167
Chesapeake Bay Retriever	1009	
Chihuahua	9	
Chinese Crested	1844	
Cockapoo	239	
Cocker Spaniel (American)	664	
Collie	7	3178
Coton de Tulear	5	
Dwarf Poodle	882	
English Cocker Spaniel	11670	
English Shepherd	8	5
Entlebucher Mountain Dog	1206	
Field Spaniel	1	
Finnish Lapphund	1938	
German Spitz	7	
Giant Schnauzer	107	
Golden Retriever	4337	
Goldendoodle	262	
Hokkaido dog		9
Karelian Bear Dog	9	
Kuvasz	531	
Lab/Golden Cross	149	
Labradoodle	1015	
Labradoodle Goldendoodle Cross	3	

Labradoodle, Australian	1822	
Labrador Retriever	25382	1
Lagotto Romagnolo	1	
Lancashire Heeler		270
Lapponian Herder	370	
Maltipoo	2	
Markiesje	1	
Miniature American Shepherd	115	37
Miniature Poodle	6519	1
Mixed Breed	2	1
Moyen/Klein Poodle	178	
Norwegian Elkhound	220	
Nova Scotia Duck Tolling Retriever	4143	2436
Pomeranian	1	
Portuguese Water Dog	4720	
Rat Terrier	1	
Schipperke	10	
Shetland Sheepdog	1	2954
Silken Windhound		118
Silky Terrier	29	
Spanish Water Dog	767	
Sprocker Spaniel	3	
Standard Poodle	348	
Swedish Lapphund	1206	
Tibetan Terrier	1	
Toy Poodle	6743	
Whippet, Longhaired		53
Yorkshire Terrier	317	

Table S5. Breeds and breed mixes emphasized in paper that met inclusion criteria of at least 50 dogs tested per year for at least eight years

**CEA**	**prcd-PRA**
Australian Shepherd	American Eskimo Dog
Border Collie	Australian Cattle Dog
Collie	Australian Shepherd
Nova Scotia Duck Tolling Retriever	Chesapeake Bay Retriever
Shetland Sheepdog	Chinese Crested
	Dwarf Poodle
	English Cocker Spaniel
	Entlebucher Mountain Dog
	Finnish Lapphund
	Golden Retriever
	Labradoodle, Australian
	Labrador Retriever
	Miniature Poodle
	Nova Scotia Duck Tolling Retriever
	Portuguese Water Dog
	Spanish Water Dog
	Swedish Lapphund
	Toy Poodle

Table S6. Breeds and breed mixes in dataset with disease-associated allele

**prcd-PRA**	**CEA**
American Cocker Spaniel	Australian Shepherd
American Eskimo Dog	Australian Shepherd, Miniature
Australian Cattle Dog	Bearded Collie
Australian Labradoodle	Berger d'Auvergne
Australian Shepherd	Border Collie
Australian Shepherd, Miniature	Boykin Spaniel
Australian Shepherd, Toy	Collie
Australian Stumpy Tail Cattle Dog	English Shepherd
Barbet	Hokkaido Dog
Bolognese	Lancashire Heeler
Bolonka Zwetna	Longhaired Whippet
Chesapeake Bay Retriever	Miniature American Shepherd
Chinese Crested	Nova Scotia Duck Tolling Retriever
Cockapoo	Shetland Sheepdog
Coton de Tulear	Silken Windhound
Dwarf Poodle	
English Cocker Spaniel	
Entlebucher Mountain Dog	
Finnish Lapphund	
Giant Schnauzer	
Golden Retriever	
Goldendoodle	
Karelian Bear Dog	
Kuvasz	
Labradoodle	
Labrador Retriever	
Labrador/Golden Retriever Cross	
Lapponian Herder	
Miniature American Shepherd	
Miniature Poodle	
Moyen/Klein Poodle	
Norwegian Elkhound	
Nova Scotia Duck Tolling Retriever	
Portuguese Water Dog	
Schipperke	
Silky Terrier	
Spanish Water Dog	
Standard Poodle	
Swedish Lapphund	
Toy Poodle	
Yorkshire Terrier	

Table S7. Relative rank of dog breeds and breed mixes comparing prcd-PRA affected to unaffected status (LRM) and affected or carrier to clear status (MRM) according to model-generated measure of likelihood (log-odds ratio) from greatest likelihood to least in models with global dataset including breed, country, and year variables

Rank	LRM for affected Genotype	LRM p-value	MRM for affected Genotype	MRM affected SE	MRM for carrier Genotype	MRM carrier SE
1	Karelian Bear Dog	0.045548	Karelian Bear Dog	5.52e-06	Karelian Bear Dog	1.33e-05
2	Barbet	0.064252	Swedish Lapphund	0.000703063	Swedish Lapphund	0.004299618
3	Swedish Lapphund	4.65e-05	Barbet	1.69e-06	American Eskimo Dog*	3.057e-05
4	Australian Stumpy Tail Cattle Dog	0.933498	Entlebucher Mountain Dog	0.00029565	Entlebucher Mountain Dog	1.865e-03
5	American Eskimo Dog*	0.921564	American Eskimo Dog*	1.60e-06	Bolognese	4.463e-05
6	Entlebucher Mountain Dog	0.97598	Australian Cattle Dog	0.001073267	Nova Scotia Duck Tolling Retriever	1.626e-02
7	Australian Cattle Dog	0.955735	Kuvasz	4.62e-05	Australian Cattle Dog	6.342e-03
8	Bolognese	0.978623	Australian Stumpy Tail Cattle Dog	7.12e-06	Kuvasz	5.781e-04
9	Cockapoo	0.584088	English Cocker Spaniel	0.002298848	Australian Shepherd, Toy	4.641e-05
10	Kuvasz	0.214248	Bolognese	1.50e-06	English Cocker Spaniel	2.005e-02
11	English Cocker Spaniel	0.006659	Australian Shepherd, Miniature	2.01e-05	Australian Stumpy Tail Cattle Dog	3.059e-05
12	Yorkshire Terrier	0.445787	Australian Shepherd, Toy	3.39e-06	Australian Shepherd, Miniature	2.254e-04
13	Australian Shepherd, Miniature	0.152148	Lapponian Herder	1.55e-05	Labradoodle, Australian	1.363e-03
14	Cocker Spaniel (American)	0.011636	Nova Scotia Duck Tolling Retriever	0.000628771	Dwarf Poodle	8.613e-04
15	Australian Shepherd, Toy	0.264379	Cockapoo	1.583e-05	Finnish Lapphund	1.702e-03
16	Giant Schnauzer	0.225084	Cocker Spaniel (American)	1.996e-05	Chesapeake Bay Retriever	6.065e-04
17	Spanish Water Dog	0.000362	Spanish Water Dog	2.990e-05	Spanish Water Dog	6.533e-04
18	Lapponian Herder	0.00393	Finnish Lapphund	7.442e-05	Norwegian Elkhound	4.329e-04

19	Finnish Lapphund	4.75e-10	Yorkshire Terrier	5.872e-06	Coton de Tulear	2.010e-06
20	Nova Scotia Duck Tolling Retriever	<2e-16	Toy Poodle	1.058e-03	Giant Schnauzer	4.828e-05
21	Toy Poodle	<2e-16	Chesapeake Bay Retriever	2.371e-05	Cockapoo	3.123e-04
22	Labradoodle	5.92e-09	Labrador Retriever	6.113e-04	Bolonka Zwetna	2.643e-05
23	Chesapeake Bay Retriever	4.31e-08	Miniature Poodle	7.277e-04	Miniature American Shepherd	4.019e-05
24	Labrador Retriever	<2e-16	Labradoodle	2.630e-05	Portuguese Water Dog	8.761e-03
25	Miniature Poodle	<2e-16	Giant Schnauzer	2.065e-06	Toy Poodle	2.562e-02
26	Miniature American Shepherd	0.103473	Dwarf Poodle	1.075e-05	Miniature Poodle	2.593e-02
27	Labrador/Golden Retriever Cross	0.01958	Portuguese Water Dog	1.544e-04	Moyen Poodle (Klein Poodle)	6.815e-05
28	Dwarf Poodle	3.16e-06	Miniature American Shepherd	9.309e-07	Labrador Retriever	1.638e-02
29	Portuguese Water Dog	<2e-16	Labrador/Golden Retriever Cross	2.296e-06	Yorkshire Terrier	1.484e-04
30	Standard Poodle	0.005655	Labradoodle, Australian	2.312e-05	Lapponian Herder	2.458e-04
31	Chinese Crested	<2e-16	Australian Cobberdog	4.091e-09	Labradoodle	5.565e-04
32	Labradoodle, Australian	4.71e-13	Markiesje	4.091e-09	Cocker Spaniel (American)	2.380e-04
33	Goldendoodle	0.008673	Chinese Crested	1.439e-05	Goldendoodle	9.412e-05
34	Golden Retriever	<2e-16	Lagotto Romagnolo	2.138e-09	Schipperke	6.888e-06
35	Australian Shepherd	1.64e-09	Border Collie	2.287e-09	Barbet	5.233e-06
36	Pomeranian	0.995835	Pomeranian	3.475e-09	Silky Terrier	7.914e-06
37	Lagotto Romagnolo	0.995795	Standard Poodle	1.231e-06	Labrador/Golden Retriever Cross	7.258e-05
38	Chihuahua	0.987674	Goldendoodle	1.670e-06	Golden Retriever	1.280e-03
39	Rat Terrier	0.995731	Field Spaniel	3.500e-09	Chinese Crested	5.514e-04
40	Border Collie	0.995711	Tibetan Terrier	3.500e-09	Standard Poodle	7.453e-05
41	Coton de Tulear	0.990626	Rat Terrier	3.921e-09	Australian Shepherd	2.257e-04
42	Black Russian Terrier	0.994851	American Hairless (Rat) Terrier	4.299e-09	Australian Cobberdog	8.001e-09
43	Maltipoo	0.993786	Boykin Spaniel	4.490e-09	Markiesje	8.001e-09

44	Australian Cobberdog	0.995663	Australian Shepherd	4.887e-06	Lagotto Romagnolo	5.103e-09
45	Field Spaniel	0.995662	Shetland Sheepdog	4.171e-09	Border Collie	5.397e-09
46	Markiesje	0.995663	Golden Retriever	7.927e-06	Pomeranian	1.358e-08
47	Tibetan Terrier	0.995662	Black Russian Terrier	8.998e-10	Field Spaniel	1.399e-08
48	Sprocker Spaniel	0.992437	Maltipoo	1.185e-09	Tibetan Terrier	1.399e-08
49	Bolonka Zwetna	0.972517	Mixed Breed	1.552e-09	Rat Terrier	2.558e-09
50	American Hairless (Rat) Terrier	0.995606	Sprocker Spaniel	5.591e-10	Shetland Sheepdog	1.122e-08
51	German Spitz	0.989962	Labradoodle, Goldendoodle Cross	8.486e-10	American Hairless (Rat) Terrier	2.785e-09
52	Boykin Spaniel	0.995564	Coton de Tulear	4.717e-10	Boykin Spaniel	2.864e-09
53	Schipperke	0.988248	Collie	2.083e-10	Black Russian Terrier	4.225e-09
54	Moyen Poodle (Klein Poodle)	0.946985	German Spitz	1.867e-10	Maltipoo	3.614e-09
55	English Shepherd	0.988952	Chihuahua	1.417e-10	German Spitz	1.762e-08
56	Labradoodle Goldendoodle Cross	0.991843	Schipperke	1.446e-10	Mixed Breed	3.733e-09
57	Mixed Breed	0.99304	English Shepherd	1.000e-10	Sprocker Spaniel	7.500e-09
58	Silky Terrier	0.981046	Silky Terrier	3.128e-10	English Shepherd	1.694e-08
59	Collie	0.991013	Bolonka Zwetna	2.271e-10	Collie	5.928e-09
60	Shetland Sheepdog	0.995151	Moyen Poodle (Klein Poodle)	7.703e-10	Labradoodle Goldendoodle Cross	2.046e-09
61	Norwegian Elkhound	0.95024	Norwegian Elkhound	3.614e-09	Chihuahua	5.602e-08

LRM = logistic regression model, MRM = multinomial regression model, SE = standard error

*Intercept in regression models

Table S8. Breeds in prcd-PRA dataset ranked by number of new dogs registered to AKC in 2004[1]

**2004 Rank**	**Breed**	**# New Registrations**
1	Labrador Retriever	146,692
2	Golden Retriever	52,550
5	Yorkshire Terrier	43,522
8	Poodle	32,671
10	Chihuahua	24,850
13	Pomeranian	21,269
15	American Cocker Spaniel	18,553
18	Shetland Sheepdog	15,605
34	Australian Shepherd	6,116
36	Collie	5,485
46	Chesapeake Bay Retriever	3,454
57	Chinese Crested	2,314
60	Border Collie	1,983
69	Portuguese Water Dog	1,535
71	Silky Terrier	1,503
74	Australian Cattle Dog	1,478
75	English Cocker Spaniel	1,326
79	Schipperke	1,092
81	Giant Schnauzer	1,012
92	Norwegian Elkhound	738
96	Tibetan Terrier	685
98	Black Russian Terrier	656
110	American Eskimo Dog	470
118	Nova Scotia Duck Tolling Retriever	318
128	Kuvasz	177
138	Field Spaniel	125

[1]Adapted from AKC registration statistics 1991-2008 (akc.org/about/archive/digital-collections/)

Table S9. Relative rank of AKC groups comparing prcd-PRA affected to unaffected status (LRM) and affected or carrier to clear status (MRM) according to model-generated measure of likelihood (log-odds ratio) from greatest likelihood to least in models with global dataset including AKC group and year variables.

Rank	LRM for affected Genotype	LRM p-value	MRM for affected Genotype	MRM affected SE	MRM for carrier Genotype	MRM carrier SE
1	Foundation Stock Service*	<2e-16	Foundation Stock Service*	9.177e-07	Foundation Stock Service*	2.214e-05
2	Herding	1.82e-05	Herding	1.578e-03	Hound	1.453e-04
3	Non-sporting	<2e-16	Non-sporting	1.143e-03	Herding	1.995e-02
4	Sporting	<2e-16	Sporting	1.022e-03	Non-sporting	2.114e-02
5	Toy	<2e-16	Toy	7.398e-04	Sporting	1.320e-02
6	Working	<2e-16	Working	5.736e-04	Working	2.583e-02
7	Terrier	0.986	Terrier	5.665e-11	Toy	2.195e-02
8	Hound	0.909	Hound	NaN	Terrier	1.373e-10

LRM = logistic regression model, MRM = multinomial regression model, SE = standard error

*Intercept in regression model

Table S10. Relative rank of FCI groups comparing prcd-PRA affected to unaffected status (LRM) and affected or carrier status (MRM) according to model-generated measure of likelihood (log-odds ratio) from greatest likelihood to least according to models with global dataset including FCI group and year variables.

**Rank**	**LRM for affected Genotype**	**LRM p-value**	**MRM for affected Genotype**	**MRM affected SE**	**MRM for carrier Genotype**	**MRM carrier SE**
1	Pinscher and Schnauzer – Molossoid and Swiss Mountain and Cattledogs	<2e-16	Pinscher and Schnauzer – Molossoid and Swiss Mountain and Cattledogs	1.068e-06	Pinscher and Schnuazer – Molossoid and Swiss Mountain and Cattledogs	1.923e-03
2	Sheepdogs and Cattledogs (except Swiss Cattledogs)	<2e-16	Sheepdogs and Cattledogs (except Swiss Cattledogs)	2.324e-03	Spitz and Primitive Types	1.657e-02
3	Terriers	0.00302	Spitz and Primitive Types	1.180e-03	Sheepdogs and Cattledogs (except Swiss Cattledogs)	2.498e-02
4	Spitz and Primitive Types	3.75e-16	Terriers	6.800e-06	Retrievers – Flushing Dogs – Water Dogs	1.566e-02
5	Retrievers – Flushing Dogs – Water Dogs	2.69e-11	Retrievers – Flushing Dogs – Water Dogs	9.644e-04	Companion and Toy Dogs*	1.874e-05
6	Companion and Toy Dogs*	<2e-16	Companion and Toy Dogs*	1.068e-06	Terriers	2.227e-04

LRM = logistic regression model, MRM = multinomial regression model, SE = standard error

*Intercept in regression equation

Table S11. Relative rank of clades comparing prcd-PRA affected to unaffected status (LRM) and affected or carrier to clear status (MRM) according to model-generated measure of likelihood (log-odds ratio) from greatest likelihood to least according to models with global dataset including clade and year variables

**Rank**	**LRM for affected Genotype**	**LRM p-value**	**MRM for affected Genotype**	**MRM affected SE**	**MRM for carrier Genotype**	**MRM carrier SE**
1	Toy Spitz	0.33	Toy Spitz	3.300e-04	Toy Spitz	1.752e-03
2	Alpine*	<2e-16	Alpine*	2.656e-07	Alpine*	1.016e-05
3	Spaniel	0.36	Spaniel	1.874e-03	Mediterranean	4.136e-04
4	UK Rural	0.17	Mediterranean	3.423e-05	Spaniel	1.713e-02
5	Mediterranean	0.23	UK Rural	1.594e-03	UK Rural	1.964e-02
6	Terrier	0.377	Terrier	4.679e-06	Unclustered1[1]	1.723e-03
7	Drover	0.342	Drover	1.427e-06	Drover	5.919e-05
8	Unclustered1[1]	9.63e-11	Unclustered1[1]	4.949e-05	Poodle	1.599e-02
9	Poodle	<2e-16	Poodle	5.996e-04	Nordic Spitz	3.640e-04
10	Retriever	<2e-16	Retriever	6.083e-04	Retriever	1.381e-02
11	Nordic Spitz	1.65e-05	Nordic Spitz	6.657e-06	Terrier	1.491e-04
12	American Toy	1.04e-14	American Toy	8.912e-06	American Toy	6.976e-04
13	Pointer Setter	0.969	Pointer Setter	9.581e-11	Pointer Setter	4.004e-10
14	Unclustered2[2]	0.968	Unclustered2[2]	1.051e-10	Unclustered2[2]	4.056e-10
15	American Terrier	0.955	American Terrier	7.476e-11	American Terrier	7.147e-11

LRM = logistic regression model, MRM = multinomial regression model, SE = standard error

*Intercept in regression equation

[1]Finnish Lapphund, did not cluster with other clades

[2]Tibetan Terrier, did not cluster with other clades

Table S12. Relative rank of all breeds and breed mixes comparing CEA affected to unaffected status (LRM) and affected or carrier to clear status (MRM) according to model-generated measure of likelihood (log-odds ratio) from greatest likelihood to least according to models with global dataset including breed, country, and year variables.

Rank	LRM for affected Genotype	LRM p-value	MRM for affected Genotype	MRM affected SE	MRM for carrier Genotype	MRM carrier SE
1	Hokkaido Dog	7.40e-09	Hokkaido Dog	5.665e-05	Hokkaido Dog	5.911e-05
2	Collie	<2e-16	Collie	1.633e-02	Collie	2.571e-02
3	Boykin Spaniel	4.04e-14	Boykin Spaniel	2.218e-04	Whippet, Longhaired	2.872e-04
4	Silken Windhound	1.68e-10	Whippet, Longhaired	9.438e-05	Shetland Sheepdog	2.396e-02
5	Whippet, Longhaired	9.34e-06	Silken Windhound	1.396e-04	Berger d'Auvergne	7.091e-05
6	Shetland Sheepdog	<2e-16	Shetland Sheepdog	8.510e-03	Boykin Spaniel	1.258e-03
7	Border Collie	0.00067	Border Collie	8.850e-03	Silken Windhound	7.171e-04
8	Bearded Collie	0.778	Bearded Collie	4.244e-05	Border Collie	2.293e-02
9	Australian Shepherd*	0.96233	Australian Shepherd*	5.506e-06	English Shepherd	2.159e-05
10	Lancashire Heeler	0.81349	Lancashire Heeler	2.357e-05	Lancashire Heeler	1.452e-03
11	Nova Scotia Duck Tolling Retriever	0.38093	Nova Scotia Duck Tolling Retriever	3.634e-04	Nova Scotia Duck Tolling Retriever	3.209e-02
12	Miniature Poodle	0.99633	Miniature Poodle	2.151e-08	Australian Shepherd*	3.693e-05
13	Mixed Breed	0.99633	Mixed Breed	2.151e-08	Bearded Collie	8.588e-04
14	English Shepherd	0.99267	Labrador Retriever	1.883e-08	Miniature American Shepherd	1.060e-05
15	Australian Shepherd, Toy	0.98586	English Shepherd	1.583e-08	Australian Shepherd, Miniature	4.883e-05
16	Labrador Retriever	0.99601	Australian Shepherd, Toy	1.437e-08	Miniature Poodle	1.814e-08
17	Miniature American Shepherd	0.98058	Berger d'Auvergne	1.071e-08	Mixed Breed	1.814e-08
18	Australian Shepherd, Miniature	0.95199	Miniature American Shepherd	1.402e-08	Labrador Retriever	2.802e-08
19	Berger d'Auvergne	0.99057	Australian Shepherd, Miniature	NaN	Australian Shepherd, Toy	1.870e-08

LRM = logistic regression model, MRM = multinomial regression model, SE = standard error

*Intercept in regression models

Table S13. Breeds in CEA dataset ranked by number of new dogs registered to AKC in 2005[1]

2005 Rank	Breed	# New Registrations
1	Labrador Retriever	137,867
8	Poodle	31,638
18	Shetland Sheepdog	14,278
34	Australian Shepherd	6,221
36	Collie	5,098
55	Border Collie	2,378
101	Bearded Collie	485
113	Nova Scotia Duck Tolling Retriever	347

[1]Adapted from AKC registration statistics 1991-2008 (akc.org/about/archive/digital-collections/)

Table S14. Breeds in prcd-PRA and CEA dataset ranked by number of new dogs registered to AKC in 2019[1]

**2019 Rank**	**Breed**
1	Labrador Retriever
3	Golden Retriever
6	Poodle
12	Yorkshire Terrier
13	Australian Shepherd
24	Pomeranian
25	Shetland Sheepdog
28	American Cocker Spaniel
29	Miniature American Shepherd
33	Border Collie
35	Chihuahua
38	Collie
46	Chesapeake Bay Retriever
49	Portuguese Water Dog
50	English Cocker Spaniel
55	Australian Cattle Dog
72	Giant Schnauzer
81	Chinese Crested
82	Coton de Tulear
87	Nova Scotia Duck Tolling Retriever
90	Boykin Spaniel
91	Lagotto Romagnolo
92	Rat Terrier
94	Norwegian Elkhound
95	Barbet
103	Tibetan Terrier
108	Schipperke
111	Silky Terrier
116	Black Russian Terrier
122	American Eskimo Dog
126	American Hairless (Rat) Terrier
138	Bearded Collie
146	Spanish Water Dog
155	Field Spaniel
167	Finnish Lapphund
168	Kuvasz
169	Entlebucher Mountain Dog

[1]adapted from AKC 2019 Most Popular Dog Breeds (based on most recent publicly available rankings; registration counts not available) (https://www.akc.org/most-popular-breeds/2019-full-list/)

Table S15. Relative rank of AKC groups comparing CEA affected to unaffected status (LRM) and affected or carrier to clear status (MRM) according to model-generated measure of likelihood (log-odds ratio) from greatest likelihood to least according to models with global dataset including AKC group and year variables

**Rank**	**LRM for -/-**	**LRM p-value**	**MRM for -/-**	**MRM -/- SE**	**MRM for +/-**	**MRM +/- SE**
1	Foundation Stock Service*	0.235	Foundation Stock Service*	1.644e-07	Foundation Stock Service*	6.138e-06
2	Herding	3.29e-05	Herding	3.374e-04	Herding	2.522e-02
3	Sporting	1.06e-10	Sporting	2.789e-04	Miscellaneous	1.408e-03
4	Miscellaneous	9.62e-06	Miscellaneous Class	8.342e-06	Sporting	2.376e-02
5	Non-sporting	0.931	Non-sporting	3.023e-11	Non-sporting	2.726e-10

LRM = logistic regression model, MRM = multinomial regression model, SE = standard error

*Intercept in regression models

Table S16. Relative rank of FCI groups comparing CEA affected to unaffected status (LRM) and affected or carrier to clear status (MRM) according to model generated measure of likelihood (log-odds ratio) from greatest likelihood to least according to models with global dataset including FCI group and year variables

**Rank**	**LRM for affected Genotype**	**LRM p-value**	**MRM for affected Genotype**	**MRM affected SE**	**MRM for carrier Genotype**	**MRM carrier SE**
1	Spitz and Primitive Types	0.931	Spitz and Primitive Types	6.417e-05	Spitz and Primitive Types	6.418e-05
2	Sheepdogs and Cattledogs (except Swiss Cattledogs)	0.95	Companion and Toy Dogs*	2.278e-07	Sheepdogs and Cattledogs (except Swiss Cattledogs)	2.624e-02
3	Retrievers – Flushing Dogs – Water Dogs	0.965	Sheepdogs and Cattledogs (except Swiss Cattledogs)	2.325e-04	Retrievers – Flushing Dogs – Water Dogs	2.618e-02
4	Companion and Toy Dogs*	0.83	Retrievers – Flushing Dogs – Water Dogs	1.680e-04	Companion and Toy Dogs*	8.792e-06

LRM = logistic regression model, MRM = multinomial regression model, SE = standard error

*Intercept in regression models

Table S17. Relative rank of clades comparing CEA affected to unaffected status (LRM) and affected or carrier to clear status (MRM) according to model-generated measure of likelihood (log-odds ratio) from greatest likelihood to least according to models with global dataset including clade and year variables

Rank	LRM for affected Genotype	LRM p-value	MRM for affected Genotype	MRM affected SE	MRM for carrier genotype	MRM carrier SE
1	Spaniel	0.948	Spaniel	1.518e-04	Spaniel	1.350e-03
2	UK Rural	0.95	Poodle	3.489e-07	UK Rural	2.658e-02
3	Retriever	0.965	UK Rural	3.130e-04	Retriever	2.523e-02
4	Poodle*	0.849	Retriever	1.608e-04	Poodle	6.644e-06

LRM = logistic regression model, MRM = multinomial regression model, SE = standard error

*Intercept in regression models

Table S18. CHIC* enforcement of genetic testing for prcd-PRA and CEA in breeds included in dataset

	prcd-PRA		CEA	
**Breed**	**Optional**	**Required**	**Optional**	**Required**
American Eskimo Dog		√		
American Hairless (Rat) Terrier	√			
Australian Cattle Dog		√		
Australian Shepherd			√	
Australian Shepherd, Miniature[1]			√	
Barbet		√		
Bearded Collie[2]				
Black Russian Terrier[2]				
Border Collie			√	
Boykin Spaniel				√
Chesapeake Bay Retriever[3]		√		
Chihuahua[2]				
Chinese Crested		√		
Cocker Spaniel (American)[2]				
Collie[2]				
Coton de Tulear[2]				
English Cocker Spaniel		√		
Entlebucher (Mountain Dog)		√		
Field Spaniel[2]				
Finnish Lapphund[3]		√		
Giant Schnauzer	√			
Golden Retriever[2]				
Hokkaido dog (Ken)[3]	√			√
Kuvasz[3]	√			
Labradoodle, Australian		√		
Labrador Retriever	√			
Lagotto Romagnolo[2]				
Lancashire Heeler				√
Miniature American Shepherd[1]			√	
Miniature Poodle[4]		√		
Norwegian Elkhound[3]	√			
Nova Scotia Duck Tolling Retriever[3]		√		
Pomeranian[2]				
Portuguese Water Dog[3]		√		
Rat Terrier[2]				
Schipperke[2]				
Shetland Sheepdog[3]	√		√	
Silken Windhound				√
Silky Terrier[2]				
Spanish Water Dog		√		
Standard Poodle[2,4]				
Tibetan Terrier[2]				
Toy Poodle[4]		√		
Yorkshire Terrier[2]				

* https://ofa.org/chic-programs/ (accessed 2/20/2023)

[1]Miniature American Shepherd and Miniature Australian Shepherd listed as same breed according to OFA CHIC database

[2]Breed included in CHIC, but no guidelines on CEA or prcd-PRA genetic testing

[3]CHIC references Progressive Retinal Atrophy DNA test, with no specification of what type

[4]Miniature, Standard, and Toy Poodles all listed under "Poodle" in OFA CHIC database

Table S19. Relative rank of countries comparing prcd-PRA affected to unaffected status (LRM) and affected or carrier to clear status (MRM) according to model-generated measure of likelihood (log-odds ratio) from greatest likelihood to least according to models with global dataset including breed, country, and year variables.

Rank	LRM for affected Genotype	LRM p-value	MRM for affected Genotype	MRM affected SE	MRM for carrier Genotype	MRM carrier SE
1	Netherlands Antilles	0.992486	Netherlands Antilles	8.377e-10	China	4.476e-09
2	Gibraltar	0.994706	Paraguay	6.078e-06	Dominican Republic	8.621e-09
3	New Caledonia	0.994798	Uruguay	1.785e-05	Colombia	3.814e-09
4	Paraguay	0.994882	Israel	2.650e-05	French Polynesia	5.381e-09
5	Bulgaria	0.995022	Bulgaria	2.442e-06	Singapore	1.753e-08
6	Israel	0.995087	New Caledonia	3.087e-07	Greenland	1.717e-08
7	Slovenia	0.995267	Japan	9.487e-05	Guadeloupe	8.702e-08
8	Uruguay	0.995391	Slovenia	5.244e-06	Paraguay	6.103e-06
9	Brazil	0.995447	Gibraltar	3.148e-07	South Korea	6.503e-06
10	Ukraine	0.995449	Brazil	1.166e-04	Uruguay	2.768e-05
11	Spain	0.995468	South Africa	1.440e-05	Macedonia	2.631e-06
12	Japan	0.995496	Cyprus	1.223e-05	Romania	3.966e-05
13	Greece	0.995547	Spain	1.976e-05	Taiwan	3.089e-06
14	Russia	0.995581	Australia	1.004e-04	Latvia	3.301e-05
15	Cyprus	0.995586	Ukraine	2.202e-06	Bulgaria	9.991e-06
16	Slovakia	0.995589	Greece	4.531e-06	Greece	2.949e-05
17	France	0.995595	Croatia	4.692e-06	Japan	5.744e-04
18	Italy	0.995614	Portugal	6.335e-06	Brazil	5.658e-04
19	Croatia	0.995625	Italy	8.115e-05	South Africa	1.883e-04
20	South Africa	0.995632	New Zealand	5.760e-06	Portugal	6.271e-05
21	Hungary	0.995636	Slovakia	3.972e-06	Slovenia	4.862e-05
22	Luxembourg	0.995642	France	5.589e-04	Ireland	1.992e-04
23	Australia	0.995653	Romania	4.883e-06	Norway	2.975e-03
24	Portugal	0.995667	Russia	2.730e-05	Gabon	3.454e-06

25	United Kingdom	0.99569	Luxembourg	1.594e-06	Cyprus	5.476e-05
26	Czech Republic	0.995696	Austria	4.837e-05	Russia	6.142e-04
27	Iceland	0.995701	Czech Republic	2.150e-05	Hungary	2.880e-04
28	Austria	0.995706	USA	1.242e-03	Mexico	8.296e-06
29	Poland	0.995713	United Kingdom	1.091e-03	Italy	9.683e-04
30	Romania	0.995718	Finland	3.015e-04	Estonia	8.800e-05
31	USA	0.995717	Hungary	1.596e-05	USA	1.487e-02
32	Ireland	0.995745	Norway	1.456e-04	France	5.599e-03
33	Norway	0.995753	Iceland	1.351e-05	Austria	4.979e-04
34	New Zealand	0.995758	Estonia	3.331e-06	Iceland	2.561e-04
35	Belgium	0.99578	Poland	1.335e-05	Spain	2.365e-04
36	Estonia	0.995784	Ireland	7.274e-06	Australia	1.041e-03
37	Finland	0.99579	Netherlands	9.368e-05	Israel	8.559e-05
38	Netherlands	0.995816	Germany	5.101e-04	Sweden	2.426e-02
39	Germany	0.995821	Denmark	9.022e-05	United Kingdom	2.142e-02
40	Switzerland	0.995833	Canada	2.790e-04	New Zealand	1.214e-04
41	Canada	0.995858	Switzerland	4.000e-05	Canada	7.955e-03
42	Denmark	0.995874	Belgium	1.761e-05	Finland	5.910e-03
43	Sweden	0.995928	Sweden	1.227e-03	Germany	1.066e-02
44	Malta	0.999392	Andorra*	1.597e-06	Denmark	2.731e-03
45	Chile	0.999391	Malta	1.814e-10	Switzerland	6.983e-04
46	Dominican Republic	0.999567	Dominican Republic	4.896e-12	Belgium	5.769e-04
47	Bosnia and Herzegovina	0.999701	Colombia	5.403e-12	Luxembourg	1.904e-05
48	China	0.999762	China	4.298e-12	Czech Republic	3.352e-04
49	Macedonia	0.99976	French Polynesia	1.040e-11	Poland	2.037e-04
50	Qatar	0.999821	Chile	4.564e-10	Netherlands	2.030e-03
51	Guadeloupe	0.999798	Bosnia and Herzegovina	5.964e-10	Croatia	2.302e-05
52	Puerto Rico	0.999813	Panama	7.460e-10	Argentina	1.037e-05
53	Latvia	0.999784	Qatar	5.119e-10	Reunion	1.040e-05
54	Panama	0.999849	Greenland	6.715e-11	Lithuania	1.558e-05

55	Bermuda	0.999884	Singapore	6.525e-11	Ukraine	1.480e-05
56	Mexico	0.999934	Macedonia	1.516e-09	Slovakia	2.593e-05
57	Reunion	0.999935	Oman	7.028e-10	Serbia-Montenegro	7.569e-06
58	Oman	0.999984	Bermuda	8.082e-10	Andorra*	3.057e-05
59	French Polynesia	0.999992	Puerto Rico	5.594e-10	Malta	6.276e-09
60	Colombia	0.999998	Thailand	6.451e-10	Chile	5.076e-09
61	Andorra*	0.921564	South Korea	2.616e-09	Netherlands Antilles	3.374e-11
62	Thailand	0.99998	Taiwan	2.759e-09	Qatar	4.713e-09
63	South Korea	0.999973	Guadeloupe	3.931e-11	Bosnia and Herzegovina	1.574e-08
64	Argentina	0.999953	Trinidad and Tobago	9.844e-10	Panama	1.916e-08
65	Gabon	0.999955	Costa Rica	4.336e-10	Gibraltar	1.125e-08
66	Costa Rica	0.999956	Macau	7.274e-10	Trinidad and Tobago	6.323e-09
67	Liechtenstein	0.999942	Liechtenstein	6.958e-10	Puerto Rico	2.659e-09
68	Taiwan	0.999924	Gabon	9.595e-10	Thailand	3.009e-09
69	Lithuania	0.999887	Mexico	7.453e-10	Bermuda	6.867e-09
70	Serbia-Montenegro	0.999881	Latvia	1.179e-09	Macau	3.588e-09
71	Trinidad and Tobago	0.999871	Argentina	3.598e-10	Oman	7.649e-09
72	Singapore	0.999724	Reunion	1.320e-10	Liechtenstein	3.395e-09
73	Macau	0.999717	Serbia-Montenegro	2.643e-10	New Caledonia	1.552e-09
74	Greenland	0.999695	Lithuania	2.597e-10	Costa Rica	1.709e-09

LRM = logistic regression model, MRM = multinomial regression model, SE = standard error

*Intercept in regression equation

Table S20. Relative rank of countries comparing CEA affected to unaffected status (LRM) and affected or carrier to clear status (MRM) according to model-generated measure of likelihood (log-odds ratio) from greatest likelihood to least according to models with global dataset including breed, country, and year variables.

Rank	LRM affected Genotype	LRM p-value	MRM for affected Genotype	MRM affected SE	MRM for carrier Genotype	MRM carrier SE
1	Kuwait	0.99026	Serbia-Montenegro	3.066e-05	Norfolk Island	1.085e-08
2	Serbia-Montenegro	0.98096	Greece	1.355e-05	Belarus	1.188e-08
3	Greece	0.98123	Kuwait	4.089e-08	Bouvet Island	2.219e-08
4	Slovakia	0.98263	Slovakia	5.701e-05	Serbia-Montenegro	3.060e-05
5	Spain	0.98305	Slovenia	4.443e-05	Greece	1.354e-05
6	Iceland	0.98311	Poland	1.860e-04	Turkey	3.497e-05
7	Slovenia	0.98323	Iceland	6.163e-05	Lithuania	6.906e-06
8	Brazil	0.9833	Spain	2.672e-04	Croatia	1.479e-04
9	Latvia	0.98333	Russia	3.911e-05	Romania	8.769e-06
10	Poland	0.98334	Brazil	6.460e-04	Portugal	1.878e-04
11	Russia	0.98336	France	5.491e-03	New Caledonia	9.733e-06
12	France	0.98356	Latvia	6.287e-06	Chile	9.676e-06
13	Italy	0.98366	Italy	8.098e-04	Slovenia	1.948e-04
14	Denmark	0.98397	Croatia	1.774e-05	Poland	8.594e-04
15	Hungary	0.98401	Hungary	8.307e-05	Russia	1.788e-04
16	Australia	0.98405	Denmark	3.946e-04	Liechtenstein	9.577e-06
17	Croatia	0.9842	Portugal	1.046e-04	Iceland	1.842e-04
18	Austria	0.98423	Australia	4.565e-04	Switzerland	9.182e-03
19	Germany	0.98422	Switzerland	4.144e-03	France	2.127e-02
20	Japan	0.98437	Austria	3.555e-04	Sweden	6.625e-03
21	Luxembourg	0.98438	Germany	6.359e-03	Peru	3.501e-05
22	Switzerland	0.98439	Japan	1.969e-05	Czech Republic	1.690e-03
23	New Zealand	0.98444	New Zealand	3.116e-05	Hungary	4.387e-04
24	Netherlands	0.9846	Netherlands	3.751e-04	Reunion	1.309e-05
25	Belgium	0.98467	Canada	7.759e-04	Brazil	2.175e-03
26	Portugal	0.98473	Belgium	3.318e-04	Italy	5.058e-03

27	Canada	0.98477	Luxembourg	3.473e-05	Canada	7.618e-03
28	Sweden	0.98492	Sweden	3.154e-04	USA	2.871e-02
29	Norway	0.9851	Norway	7.878e-05	Denmark	2.364e-03
30	United Kingdom	0.98522	USA	4.803e-03	Finland	5.568e-03
31	Ireland	0.98531	Czech Republic	7.986e-05	Austria	2.324e-03
32	USA	0.98533	United Kingdom	1.909e-03	Netherlands	5.499e-03
33	Czech Republic	0.9854	Ireland	1.830e-04	Spain	7.128e-04
34	Finland	0.98564	Finland	3.524e-04	Germany	3.710e-02
35	Reunion	0.9984	Argentina*	5.506e-06	Australia	2.339e-03
36	Norfolk Island	0.99958	Norfolk Island	1.622e-12	Belgium	3.362e-03
37	Malta	0.99959	Belarus	4.389e-12	United Kingdom	3.114e-02
38	New Caledonia	0.99936	Malta	8.758e-10	Ireland	3.684e-03
39	Ukraine	0.99963	Ukraine	9.155e-10	Norway	2.282e-03
40	Chile	0.99939	Lithuania	1.408e-09	New Zealand	1.712e-04
41	Lithuania	0.99954	Bulgaria	1.771e-09	Japan	1.337e-04
42	Liechtenstein	0.99948	Netherlands Antilles	1.869e-09	Slovakia	1.012e-04
43	Belarus	0.99978	Romania	1.750e-09	Colombia	1.407e-05
44	Bulgaria	0.99978	Uruguay	2.027e-09	Faroe Islands	2.191e-04
45	Netherlands Antilles	0.9998	New Caledonia	1.252e-09	South Africa	5.621e-05
46	Romania	0.99972	Chile	1.362e-09	Latvia	2.128e-05
47	Uruguay	0.99982	Liechtenstein	1.489e-09	Luxembourg	8.775e-05
48	Turkey	0.99973	Bouvet Island	2.945e-11	Argentina*	3.693e-05
49	Peru	0.99987	Reunion	7.840e-10	Kuwait	6.266e-09
50	Colombia	0.99998	Turkey	1.775e-09	Ukraine	1.450e-08
51	Argentina*	0.96233	Colombia	1.893e-09	Malta	1.426e-08
52	Estonia	0.99994	Peru	1.716e-09	Bulgaria	1.430e-08
53	Faroe Islands	0.9998	Zimbabwe	1.206e-09	Netherlands Antilles	1.416e-08
54	South Africa	0.99978	Singapore	1.304e-09	Uruguay	1.394e-08
55	Mexico	0.99978	Estonia	1.391e-09	Zimbabwe	1.922e-08
56	Zimbabwe	0.99971	Mexico	1.084e-09	Singapore	1.912e-08
57	Singapore	0.99968	South Africa	1.254e-09	Mexico	1.278e-08
58	Bouvet Island	0.99912	Faroe Islands	8.658e-12	Estonia	1.071e-08

LRM = logistic regression model, MRM = multinomial regression model, SE = standard error

*Intercept in regression equation

Table S21. Relative rank of continents comparing prcd-PRA affected to unaffected status (LRM) and affected or carrier to clear status (MRM) according to model-generated measure of likelihood (log-odds ratio) from greatest likelihood to least according to models with global dataset including breed, continent, and year variables

**Rank**	**LRM for affected Genotype**	**LRM p-value**	**MRM for affected Genotype**	**MRM affected SE**	**MRM for carrier Genotype**	**MRM carrier SE**
1	Asia	0.202977	South America	9.116e-05	South America	3.836e-04
2	South America	0.179899	Asia	7.255e-05	Asia	5.775e-04
3	Oceania	0.956968	Africa*	2.850e-06	Africa*	2.760e-05
4	Africa*	< 2e-16	Oceania	1.773e-04	North America	8.006e-03
5	North America	0.575249	North America	1.960e-02	Oceania	8.833e-04
6	Europe	0.467673	Europe	1.978e-02	Europe	8.066e-03

LRM = logistic regression model, MRM = multinomial regression model, SE = standard error

*Intercept in regression equation

Table S22. Relative rank of continents comparing CEA affected to unaffected status (LRM) and affected or carrier to clear status (MRM) according to model-generated measure of likelihood (log-odds ratio) from greatest likelihood to least according to models with global dataset including breed, continent, and year variables

**Rank**	**LRM for affected Genotype**	**LRM p-value**	**MRM for affected Genotype**	**MRM affected SE**	**MRM for carrier Genotype**	**MRM carrier SE**
1	South America	0.9681	South America	7.988e-04	Antarctica	4.866e-08
2	Asia[1]	0.9694	Asia	5.587e-05	South America	1.002e-03
3	Oceania[1]	0.9694	Oceania	6.098e-04	North America	1.387e-02
4	Europe	0.9704	Europe	2.931e-02	Asia	8.506e-05
5	North America	0.9722	North America	2.841e-02	Europe	1.488e-02
6	Africa*	0.9792	Africa*	4.916e-06	Oceania	1.017e-03
7	Antarctica	0.9984	Antarctica	5.226e-13	Africa*	2.065e-05

LRM = logistic regression model, MRM = multinomial regression model, SE = standard error

*Intercept in regression equation

[1]Asia and Oceania had same relative estimate of log-odds, as well as identical p-values

Table S23. Lay public summary table of overall prcd-PRA genotype and allele frequencies and allele frequency trend direction. This does not reflect the range of sample size across time or the magnitude of trend.

**Breed/Mix**	**affected freq**	**carrier freq**	**allele freq**	**Allele freq trend**
American Eskimo Dog	0.088615	0.507629	0.34243	Decrease
American Hairless (Rat) Terrier	0	0	0	-
Australian Cattle Dog	0.065298	0.435756	0.283175	Decrease
Australian Cobberdog	0	0	0	-
Australian Shepherd	0.001834	0.03423	0.018949	Decrease
Australian Shepherd, Miniature	0.042194	0.316456	0.200422	Decrease
Australian Shepherd, Toy	0.026316	0.381579	0.217105	Decrease
Australian Stumpy Tail Cattle Dog	0.118644	0.322034	0.279661	Increase
Barbet	0.095238	0.142857	0.166667	Decrease
Black Russian Terrier	0	0	0	-
Bolognese	0.027778	0.25	0.152778	Decrease
Bolonka Zwetna	0	0.169811	0.084906	Decrease
Border Collie	0	0	0	-
Boykin Spaniel	0	0	0	-
Chesapeake Bay Retriever	0.016848	0.328048	0.180872	Increase
Chihuahua	0	0	0	Consistent
Chinese Crested	0.008677	0.125271	0.071312	Decrease
Cockapoo	0.046025	0.23431	0.16318	Decrease
Cocker Spaniel (American)	0.033133	0.185241	0.125753	Decrease
Coton de Tulear	0	0.2	0.1	Decrease
Dwarf Poodle	0.007937	0.314059	0.164966	Decrease
English Cocker Spaniel	0.053728	0.366667	0.237061	Decrease
English Shepherd	0	0	0	Consistent
Entlebucher Mountain Dog	0.077944	0.443615	0.299751	Decrease
FamCollie	0	0	0	Consistent
Field Spaniel	0	0	0	-
Finnish Lapphund	0.019092	0.326109	0.182147	Decrease
German Spitz	0	0	0	Consistent
Giant Schnauzer	0.018692	0.233645	0.135514	Decrease
Golden Retriever	0.002075	0.113904	0.059027	Decrease
Goldendoodle	0.003817	0.183206	0.09542	Decrease
Karelian Bear Dog	0.222222	0.444444	0.444444	Decrease
Kuvasz	0.050847	0.397363	0.249529	Decrease
Lab/Golden Cross	0.013423	0.161074	0.09396	Increase
Labradoodle	0.02069	0.240394	0.140887	Decrease
Labradoodle Goldendoodle Cross	0	0	0	Consistent
Labradoodle, Australian	0.006037	0.334248	0.173161	Decrease
Labrador Retriever	0.016823	0.230006	0.131826	Decrease
Lagotto Romagnolo	0	0	0	-
Lapponian Herder	0.021622	0.194595	0.118919	Increase
Maltipoo	0	0	0	Consistent

Markiesje	0	0	0	-
Miniature American Shepherd	0.008696	0.243478	0.130435	Decrease
Miniature Poodle	0.0158	0.256941	0.144271	Decrease
Mixed Breed	0	0	0	Consistent
Moyen Poodle (Klein Poodle)	0	0.235955	0.117978	Decrease
Norwegian Elkhound	0	0.359091	0.179545	Decrease
Nova Scotia Duck Tolling Retriever	0.01762	0.446778	0.241009	Decrease
Pomeranian	0	0	0	-
Portuguese Water Dog	0.009322	0.25572	0.137182	Decrease
Rat Terrier	0	0	0	-
Schipperke	0	0.1	0.05	Increase
Shetland Sheepdog	0	0	0	-
Silky Terrier	0	0.103448	0.051724	Decrease
Spanish Water Dog	0.022164	0.29987	0.172099	Increase
Sprocker Spaniel	0	0	0	Consistent
Standard Poodle	0.005747	0.08046	0.045977	Decrease
Swedish Lapphund	0.075456	0.524046	0.337479	Decrease
Tibetan Terrier	0	0	0	-
Toy Poodle	0.023283	0.274803	0.160685	Decrease
Yorkshire Terrier	0.025237	0.170347	0.11041	Increase

-only one data point, not enough data to determine a trend

Table S24. Lay public summary table of overall CEA genotype and allele frequencies and allele frequency trend direction. This does not reflect the range of sample size across time or the magnitude of trend.

**Breed/Mix**	**affected freq**	**carrier freq**	**allele freq**	**Allele freq trend**
Australian Shepherd	0.010201	0.164142	0.092272	Increase
Australian Shepherd, Miniature	0	0.005102	0.002551	Decrease
Australian Shepherd, Toy	0	0	0	Consistent
Bearded Collie	0.005634	0.08169	0.046479	Decrease
Berger d'Auvergne	0	0.444444	0.222222	Increase
Border Collie	0.013107	0.280451	0.153333	Decrease
Boykin Spaniel	0.05988	0.407186	0.263473	Increase
Collie	0.249843	0.438326	0.469006	Increase
English Shepherd	0	0.2	0.1	Increase
Hokkaido dog	0.444444	0.444444	0.666667	Decrease
Labrador Retriever	0	0	0	-
Lancashire Heeler	0.003704	0.248148	0.127778	Decrease
Miniature American Shepherd	0	0.027027	0.013514	Increase
Miniature Poodle	0	0	0	-
Mixed Breed	0	0	0	-
Nova Scotia Duck Tolling Retriever	0.004516	0.197455	0.103243	Decrease
Shetland Sheepdog	0.098849	0.422139	0.309919	Decrease
Silken Windhound	0.059322	0.338983	0.228814	Increase
Whippet, Longhaired	0.075472	0.528302	0.339623	Decrease

-only one data point, not enough data to determine a trend
