# Supplementary figures and images for: Global Frequency Analyses of Canine Progressive Rod-Cone Degeneration–Progressive Retinal Atrophy and Collie Eye Anomaly Using Commercial Genetic Testing Data
Source: Genes (Basel). 2023 Nov 17;14(11):2093. doi: 10.3390/genes14112093 (PMC10671078; doi:10.3390/genes14112093)

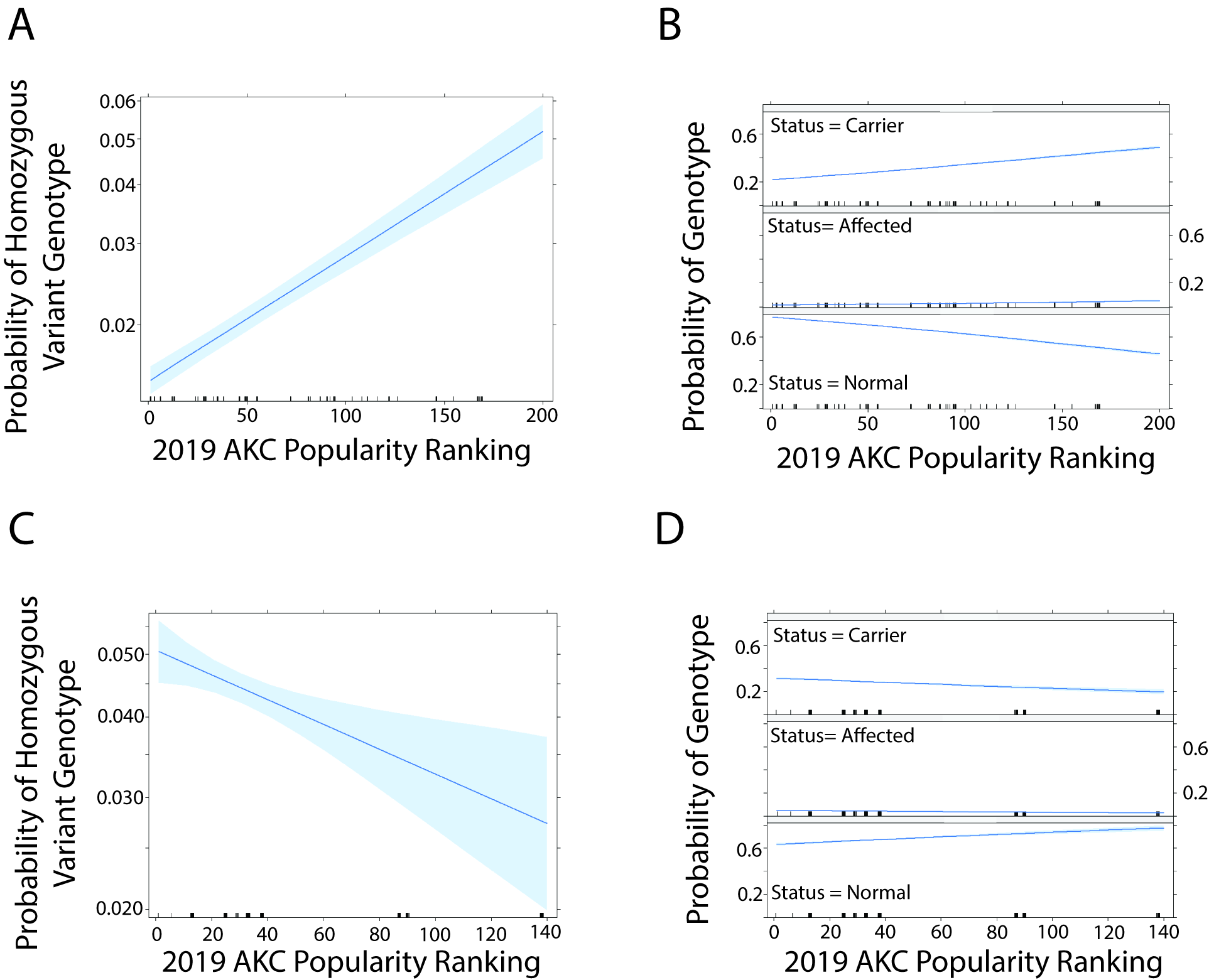

Supplement: Supplementary file 1 [file genes-14-02093-s001.zip › Supplemental Tifs for Paper/Figure S1.tif]

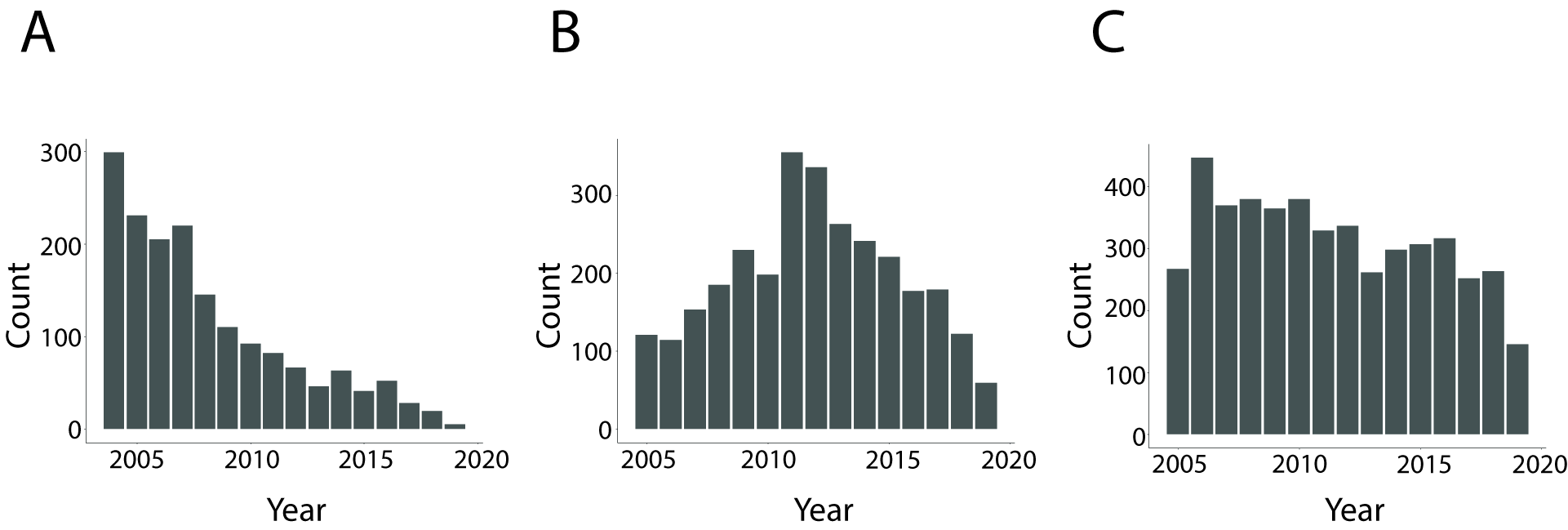

Supplement: Supplementary file 1 [file genes-14-02093-s001.zip › Supplemental Tifs for Paper/Figure S2.tif]

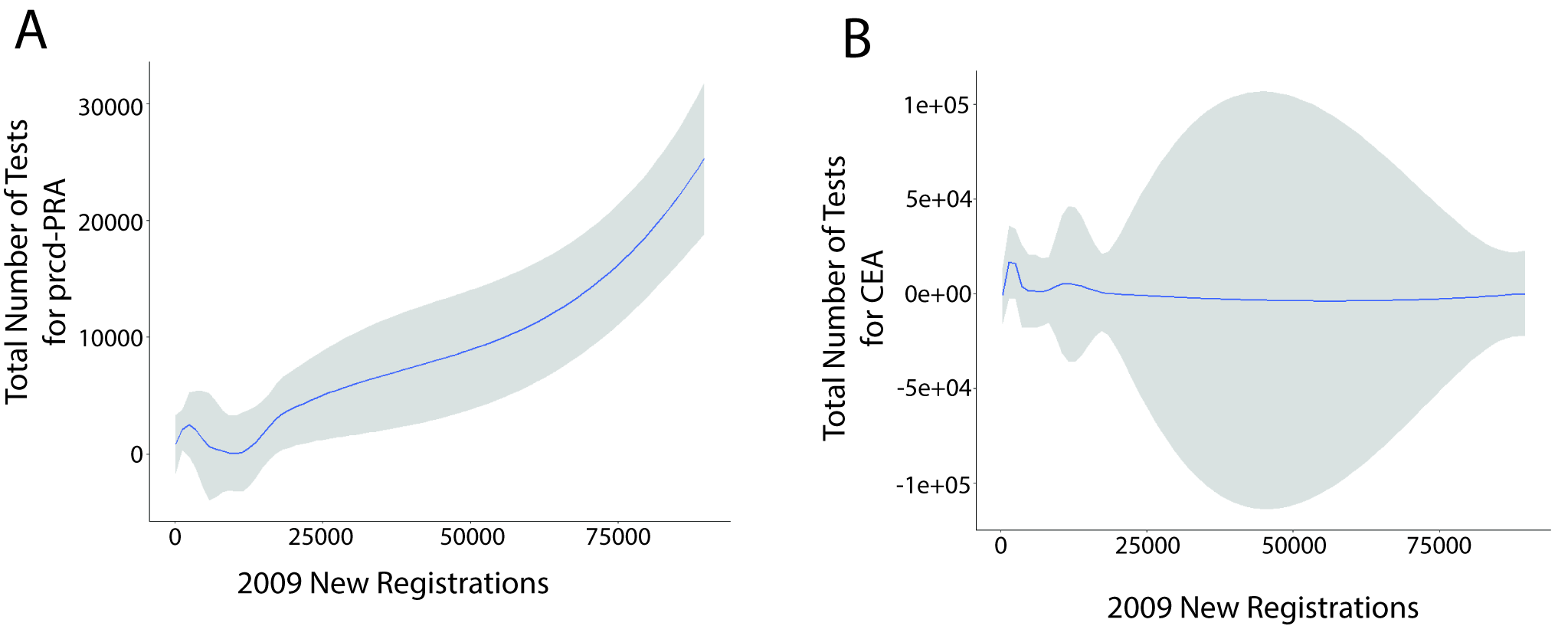

Supplement: Supplementary file 1 [file genes-14-02093-s001.zip › Supplemental Tifs for Paper/Figure S3.tif]

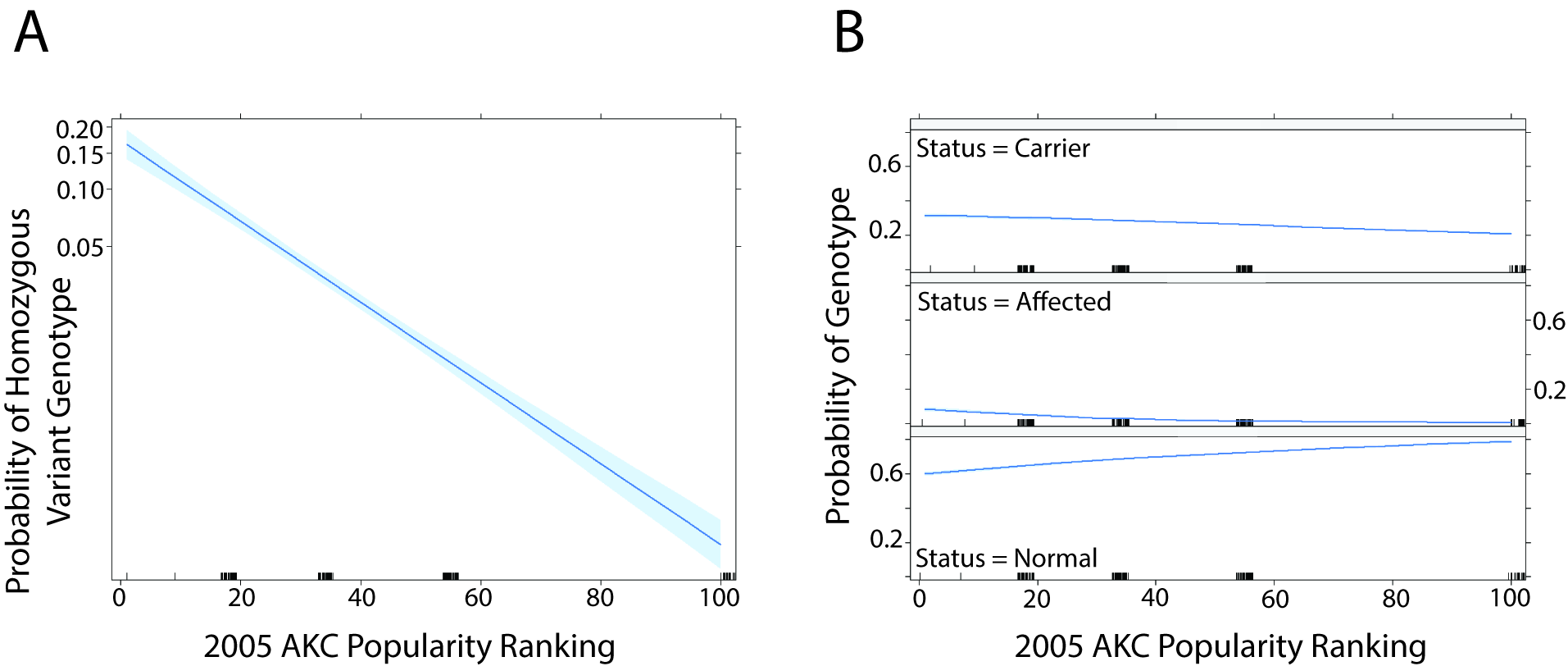

Supplement: Supplementary file 1 [file genes-14-02093-s001.zip › Supplemental Tifs for Paper/Figure S4.tif]

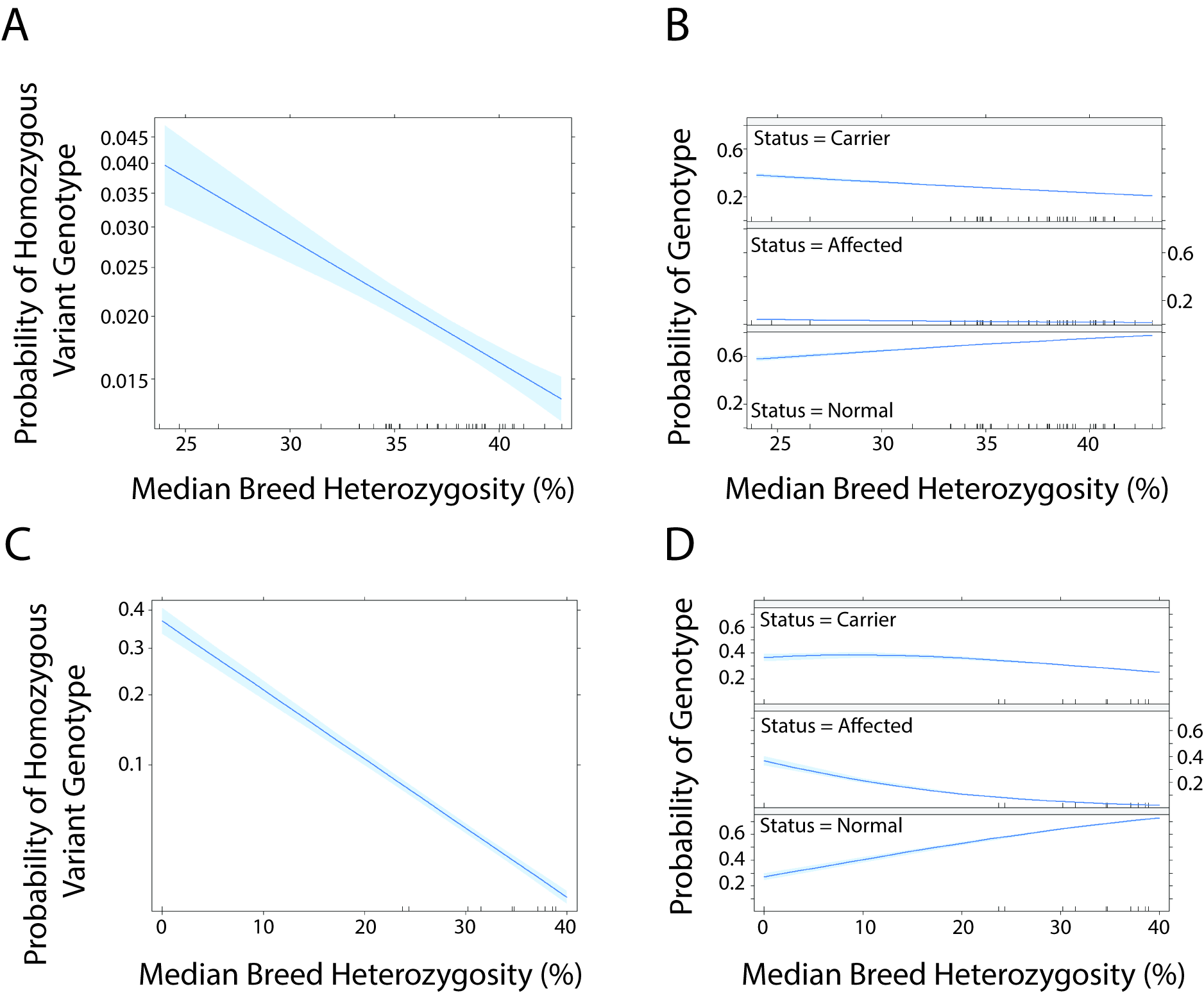

Supplement: Supplementary file 1 [file genes-14-02093-s001.zip › Supplemental Tifs for Paper/Figure S5.tif]

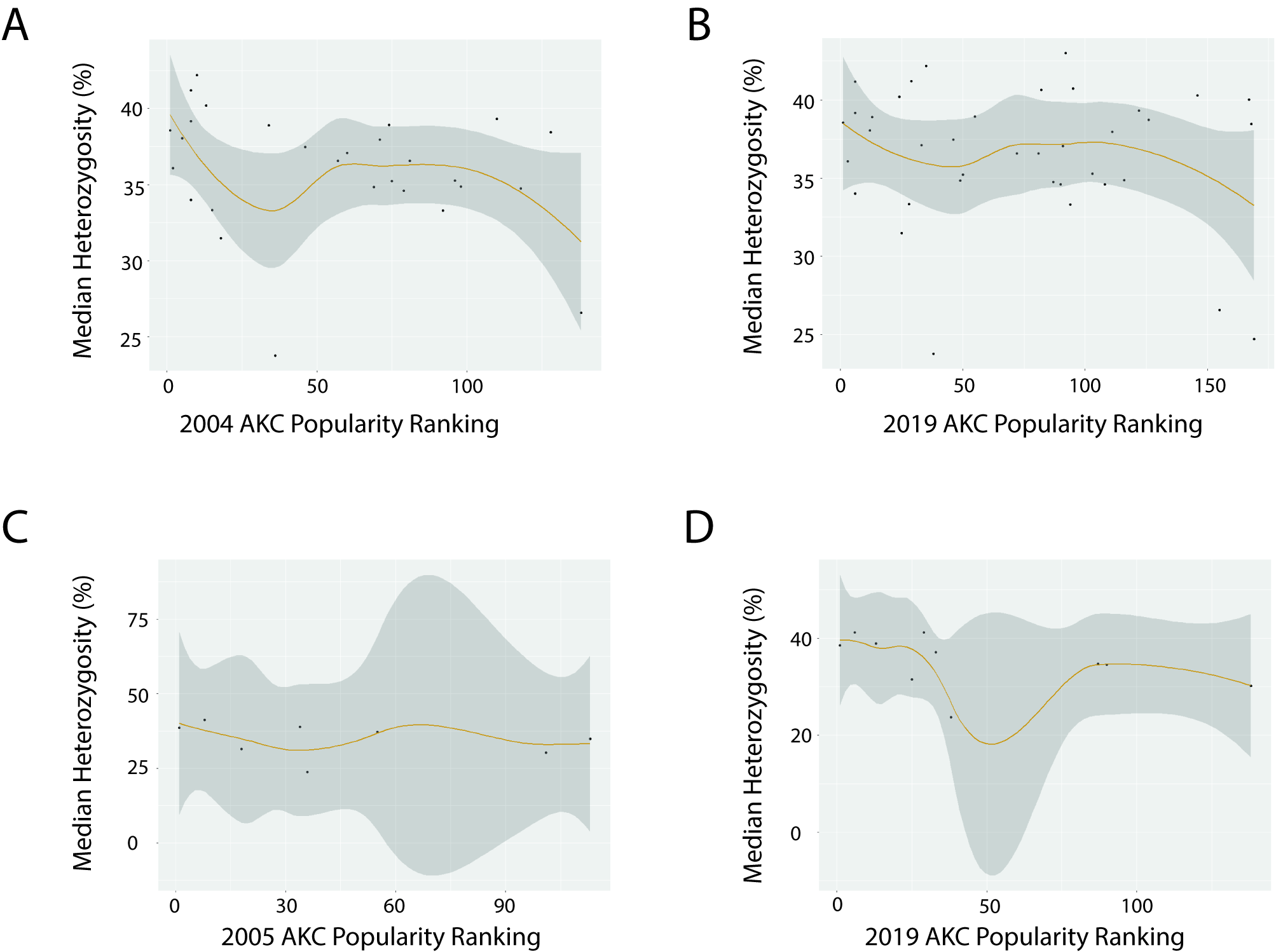

Supplement: Supplementary file 1 [file genes-14-02093-s001.zip › Supplemental Tifs for Paper/Figure S6.tif]
